# Gut microbiota and tuberculosis

**DOI:** 10.1002/imt2.70054

**Published:** 2025-06-22

**Authors:** Yanhua Liu, Ling Yang, Maryam Meskini, Anjana Goel, Monique Opperman, Sagar Singh Shyamal, Ajay Manaithiya, Meng Xiao, Ruizi Ni, Yajing An, Mingming Zhang, Yuan Tian, Shuang Zhou, Zhaoyang Ye, Li Zhuang, Linsheng Li, Istuti Saraswat, Ankita Kar, Syed Luqman Ali, Shakir Ullah, Syed Yasir Ali, Shradha Kaushik, Tianmu Tian, Mingyang Jiao, Shujun Wang, Giulia Ghisleni, Alice Armanni, Sara Fumagalli, WenYu Wang, Chao Cao, Maria Carpena, Miguel A. Prieto, Antonia Bruno, Chanyuan Jin, Hanqing Hu, Yuhang Zhang, Ilse du Preez, Ashok Aspatwar, Lingxia Zhang, Wenping Gong

**Affiliations:** ^1^ Institute of Tuberculosis, Senior Department of Tuberculosis the Eighth Medical Center of PLA General Hospital Beijing China; ^2^ Graduate School Hebei North University Zhangjiakou China; ^3^ Department of Mycobacteriology and Pulmonary Research Pasteur Institute of Iran Tehran Iran; ^4^ Microbiology Research Center (MRC) Pasteur Institute of Iran Tehran Iran; ^5^ Department of Biotechnology GLA University Mathura India; ^6^ Centre for Human Metabolomics North‐West University Potchefstroom South Africa; ^7^ Department of Pharmaceutical Engineering & Technology Indian Institute of Technology (Banaras Hindu University) Varanasi India; ^8^ Faculty of Medicine and Health Technology Tampere University Tampere Finland; ^9^ Computer Network Information Center Chinese Academy of Sciences Beijing China; ^10^ ICMR NHRP project‐Assam Medical College Dibrugarh India; ^11^ Department of Biochemistry Abdul Wali Khan University Mardan Pakistan; ^12^ Department of Zoology Abdul Wali Khan University Mardan Pakistan; ^13^ Department of Pathology Abdul Wali Khan University Mardan Pakistan; ^14^ Department of Biotechnology Institute of Engineering and Technology Lucknow India; ^15^ School of Management Science and Engineering Beijing University of Information Science and Technology Beijing China; ^16^ Department of Prosthodontics Peking University School and Hospital of Stomatology & National Health Commission Stomatology Computer Application Engineering Technology Research Center & National Center for Stomatology Beijing China; ^17^ School and Hospital of Stomatology China Medical University, Liaoning Provincial Key Laboratory of Oral Disease Shenyang China; ^18^ Biotechnology and Biosciences Department University of Milano‐Bicocca Milan Italy; ^19^ Beijing Anzhen Hospital Capital Medical University Beijing China; ^20^ Department of Respiratory and Critical Care Medicine, Key Laboratory of Respiratory Disease of Ningbo The First Affiliated Hospital of Ningbo University Ningbo China; ^21^ Universidade de Vigo, Nutrition and Food Group (NuFoG), Department of Analytical Chemistry and Food Science Instituto de Agroecoloxía e Alimentación (IAA) – CITEXVI Vigo Spain; ^22^ 2nd Dental Center Peking University School and Hospital of Stomatology Beijing China; ^23^ Institute of Clinical Pharmacology Peking University First Hospital Beijing China

**Keywords:** artificial intelligence, gut microbiota, microbiome‐based diagnostics, *Mycobacterium tuberculosis*, omics technologies, tuberculosis

## Abstract

Tuberculosis (TB), caused by *Mycobacterium tuberculosis* (MTB), remains a significant global health challenge. Recent advancements in gut microbiota (GM) research have shed light on the intricate relationship between GM and TB, suggesting that GM alterations may influence host susceptibility, disease progression, and response to antituberculosis drugs. This review systematically synthesizes and analyzes the current research progress on the relationship between GM and TB, focusing on six key aspects: (1) bidirectional effects between GM dynamics and TB progression; (2) the interaction between GM and anti‐TB drugs; (3) GM and TB immune response; (4) GM as a potential target for diagnosis and treatment of TB; (5) multi‐omics and artificial intelligence (AI) technologies in GM‐TB research; (6) current challenges and future directions in GM‐TB research. We highlight the bidirectional nature of the GM–TB interaction, where MTB infection can lead to GM dysbiosis, and changes can affect the host's immune response, contributing to TB onset and progression. Advanced molecular techniques, such as next‐generation sequencing and metagenomics, along with AI, play pivotal roles in elucidating these complex interactions. Future research directions include investigating the relationship between GM and TB vaccine efficacy, exploring GM's potential in TB prevention, developing microbiome‐based diagnostic and prognostic tools, and examining the role of GM in TB recurrence. By addressing these areas, we aim to provide a comprehensive perspective on the latest advancements in GM and TB research and offer insights for future studies and clinical applications. Ultimately, the development of novel microbiome‐based strategies may offer new tools and insights for the effective control and management of TB, a disease that continues to pose a significant threat to public health.

## Highlights

- Bidirectional gut microbiota (GM)–tuberculosis (TB) interaction reveals a dynamic interplay where *Mycobacterium tuberculosis* infection disrupts GM, while GM dysbiosis exacerbates TB progression by modulating host immunity.
- Technological innovation integrates next‐generation sequencing, metagenomics, and artificial intelligence (AI) to unravel complex GM‐TB relationships, enabling predictive modeling and precision medicine approaches.
- Regarding GM as a diagnostic/therapeutic target, researchers propose GM modulation as a novel strategy to enhance anti‐TB drug efficacy, mitigate side effects, and develop microbiome‐based diagnostics for TB susceptibility and prognosis.
- A comprehensive research framework systematically synthesizes six key areas—from GM‐immune crosstalk to recurrence mechanisms—providing a roadmap for future TB management strategies and vaccine optimization.

## Graphical abstract

This review explores the intricate relationship between gut microbiota (GM) and tuberculosis (TB). The figure illustrates the bidirectional effects between GM dynamics and TB progression, as well as the interactions between the GM and antituberculosis drugs. It also explores the role of GM in the immune response to TB and its potential as a target for the diagnosis and treatment of TB. Finally, the application of multi‐omics and artificial intelligence (AI) technologies in GM‐TB research is highlighted. The review aims to shed light on the complex interplay between GM and TB, paving the way for innovative strategies in TB management.

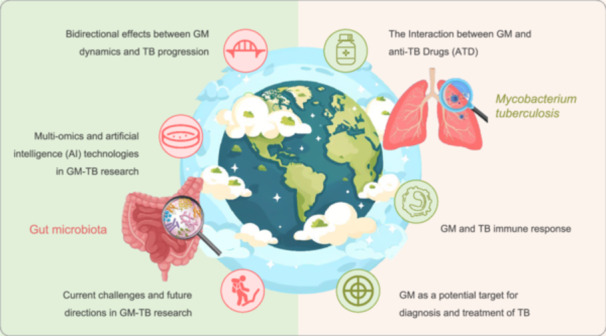

## INTRODUCTION

Tuberculosis (TB), caused by *Mycobacterium tuberculosis* (MTB), remains a formidable global health challenge despite decades of control efforts [1, 2]. As the leading infectious killer second only to Corona Virus Disease 2019, TB claimed 1.25 million lives in 2023, with 10.8 million new cases reported worldwide [3]. This persistent burden stems from complex host–pathogen interactions and socioeconomic determinants, necessitating novel therapeutic approaches [4, 5].

The gut microbiota (GM) has emerged as a pivotal regulator of systemic immunity, comprising >100 trillion microorganisms that interact with host physiology through metabolic cross‐talk and immune modulation [6, 7]. Beyond its established roles in metabolic disorders and gastrointestinal diseases [8, 9, 10], GM dysbiosis is increasingly implicated in respiratory infections through the gut–lung axis—a bidirectional communication network linking intestinal microbiota with pulmonary immunity [11, 12, 13].

Emerging evidence reveals intricate GM‐TB interplay: (1) Preclinical models demonstrate that GM‐derived metabolites like butyrate enhance macrophage antimicrobial responses via histone deacetylase inhibition [14]; (2) Clinical studies document characteristic GM alterations in TB patients, including reduced *Faecalibacterium* and elevated *Enterobacteriaceae* [14, 15, 16, 17]; (3) Anti‐TB chemotherapy induces prolonged GM perturbations, potentially compromising treatment efficacy through immunometabolic disruption [18, 19, 20, 21, 22]. Notably, Mendelian randomization analysis suggests GM composition may causally influence TB susceptibility, with specific taxa exhibiting protective or risk‐enhancing effects [23].

This evolving paradigm raises critical questions: Could GM modulation enhance the efficacy of the Bacille Calmette‐Guérin (BCG) vaccine, a widely used vaccine for TB? How do geographically distinct GM profiles influence TB epidemiology? What biomarkers predict treatment‐induced dysbiosis? Addressing these questions requires integrating multi‐omics approaches with clinical validation—a gap this review seeks to highlight.

We systematically evaluate six key aspects of GM–TB interactions: (1) bidirectional effects between GM dynamics and TB progression; (2) the interaction between GM and anti‐TB drugs (ATD); (3) GM and TB immune response; (4) GM as a potential target for diagnosis and treatment of TB; (5) multi‐omics and artificial intelligence (AI) technologies in GM‐TB research; (6) current challenges and future directions in GM‐TB research. Through an in‐depth exploration of these aspects, we hope to provide readers with a comprehensive perspective on the latest advancements in this emerging research field of GM and TB, as well as offer ideas and insights for future research and clinical applications. As our understanding of the relationship between GM and TB deepens, we anticipate the development of novel microbiome‐based diagnostic, therapeutic, and preventive strategies, providing new weapons for the control of TB.

## BIDIRECTIONAL EFFECTS BETWEEN GM DYNAMICS AND TB PROGRESSION

**FIGURE 1:**
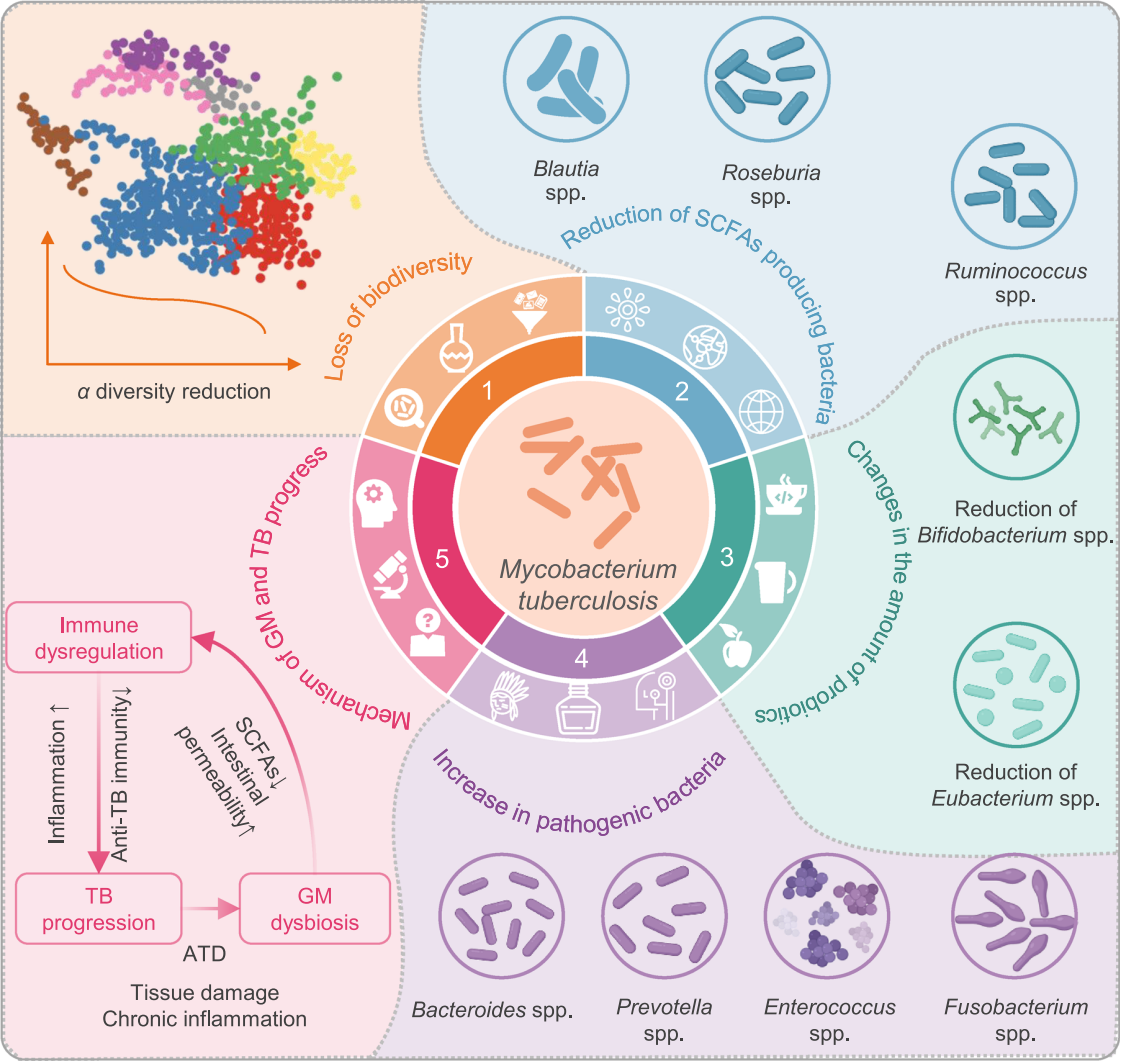
Relationship between gut microbiota (GM) dysbiosis and tuberculosis (TB) progression. The figure illustrates the association between GM dysbiosis and TB pathogenesis. Dysbiosis is characterized by a loss of biodiversity, particularly a depletion of key short‐chain fatty acid (SCFA)‐producing taxa such as *Blautia* spp., *Roseburia* spp., *Ruminococcus* spp., *Bifidobacterium* spp., and *Eubacterium* spp. This is accompanied by an overrepresentation of potentially pathogenic genera, including *Bacteroides* spp., *Prevotella* spp., *Enterococcus* spp., and *Fusobacterium* spp. These compositional shifts contribute to increased intestinal permeability, disruption of mucosal immune homeostasis, and impaired host immune responses against *Mycobacterium tuberculosis*, ultimately facilitating disease progression. ATD, antituberculosis drugs.

**FIGURE 2:**
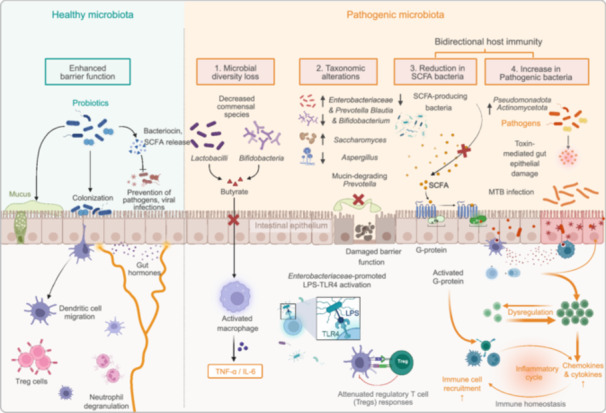
Comparative characterization of healthy versus pathogenic gut microbiota states. This figure highlights the contrasting features of a healthy versus dysbiotic gut microbiota. In healthy state, commensal bacteria such as *Lactobacilli* and *Bifidobacteria* reinforce intestinal barrier function through the production of bacteriocins and short‐chain fatty acids (SCFAs), particularly butyrate. These microbes preferentially colonize the mucosal layer, prevent pathogenic colonization, and modulate host immunity by promoting dendritic cell migration and the release of gut‐derived hormones. In pathogenic state, microbial diversity is reduced (1), with a concomitant overrepresentation of mucin‐degrading bacteria (2) and a loss of beneficial SCFA‐producing species (3). This altered microbial composition compromises intestinal epithelial integrity and enables translocation of pathogens such as *Mycobacterium tuberculosis* (MTB), which contribute to tissue damage via toxin‐mediated mechanisms (4). The reduction in SCFA‐producing bacteria and rise in pathogenic *Enterobacteriaceae* promote immune dysregulation through lipopolysaccharides (LPS)‐mediated activation of Toll‐like receptor 4 (TLR4) and G‐protein signaling. This cascade triggers a chronic inflammatory response characterized by elevated cytokine production, neutrophil degranulation, and impaired regulatory T cell function, ultimately disrupting immune homeostasis.

The systemic impact of TB extends beyond pulmonary manifestations, with growing evidence revealing profound GM alterations in TB patients compared to healthy individuals. These compositional changes encompass microbial diversity loss, phylum‐level restructuring, and functional metabolic shifts, which not only emerge as potential biomarkers for disease monitoring and therapeutic targets, but also drive disease progression through immuno–metabolic interactions (Table 1) [16, 24, 25, 26, 27, 28, 29, 30, 31]. The observed GM perturbations in TB patients are characterized by three interconnected phenomena: reduced microbial diversity, specific taxonomic alterations, and bidirectional interactions with host immunity (Figure 1). This section systematically explores the impact of GM on the immune response and barrier function, and illustrates how alterations in GM composition influence the progression of TB. As shown in Figure 2, the mechanisms underlying the bidirectional interactions between GM and TB are also highlighted, showcasing the role of both health‐promoting and pathogenic bacteria in disease progression [15, 32, 33].

**TABLE 1:** Overview of changes in GM composition in TB patients and animal models.

Year*	Species (Sample size)	Analysis methods	Diversity changes	Bacterial changes	Fungal changes	Possible impacts	Refs
2024	Human (*n* = 71, including 23 naive TB patients, 48 HCs)	Deep shotgun sequencing; Illumina HiSeq. 2500 platform; Metagenomic analysis	No significant change in α diversity, increase in β diversity	Increase in *Actinomycetota*, *Bacillota*, and *Pseudomonadota*; decrease in *Bacteroidota*	Unknown	Increased metabolic pathways related to cell division and growth affect gut health.	[16]
2023	Human (*n* = 53, including 33 TB patients, 20 HCs)	16S rRNA sequencing (V3–V4 region); ITS gene sequencing; MiSeq PE300 platform; ASVs	Significant reduction in α diversity	Significant increase in *Bacteroides* and *Prevotella*; decrease in *Blautia* and *Bifidobacterium*	Increase in *Ascomycota*; decrease in *Basidiomycota*	May influence disease pathogenesis or pathology; potential auxiliary strategy for TB treatment	[24]
2022	Human (*n* = 135, including 83 PTB patients, 52 HCs)	16S rRNA sequencing (V3–V4 region); Ion S5TMXL platform; OTUs	Significant reduction in α diversity	Increase in *Bacteroidaceae*, *Tannerellaceae*, *Fusobacteriaceae*, *Erysipelotrichaceae*; decrease in *Bifidobacteriaceae*, *Lachnospiraceae*, *Ruminococcaceae*, *Marinifilaceae*, *Eggerthellaceae*, *Barnesiellaceae*	Unknown	Changes in GM structure affecting SCFA production	[25]
2022	Human (*n* = 142, including 56 PTB patients, 36 LTBI, 50 HCs)	16S rRNA sequencing (V3‐V4 region); Illumina NovaSeq platform; ASVs	Significant reduction in α diversity	Decrease in *Bacillota*; increase in *Bacteroidota*	Unknown	Immune dysregulation leading to impaired immune response against TB	[26]
2019	Human (*n* = 77, including 46 TB patients, 31 HCs)	Shotgun metagenomic sequencing; Illumina HiSeq. 2500; Species‐level resolution	Significant reduction in α diversity and abundance	Decrease in *Bacillota* and *Bacteroidota*; increase in *Pseudomonadota*	Unknown	Potential to distinguish TB patients from non‐TB patients	[27]
2018	Human (*n* = 24, including 6 HCs, 6 TBZ, 6 TBW, 6 TBM)	16S rRNA sequencing (V3 region); Whole‐genome shotgun sequencing; Illumina HiSeq. 2000 and NextSeq. 500 platforms; OTUs and KOs	Higher α diversity and species richness	Decrease in *Prevotella*, *Faecalibacterium*, *Roseburia*, *Eubacterium*, and *Bacteroidota*; increase in *Bacillota*	Unknown	Reduced SCFA production with enriched propionate‐ and butyrate‐producing bacteria leads to impaired host immune response	[28]
2017	Mouse (*n* = 3–5/group, group = 11)	16S rRNA sequencing (V3–V4 region); Illumina MiSeq platform; ASVs	Temporary reduction in diversity but significant and persistent structural changes	Changes in *Clostridiales*, *Bacteroidota*, and *Tenericutes*	Unknown	Structural changes in GM affecting host disease resistance and immune function	[29]
2017	Human (*n* = 57, including 19 new TB patients, 18 recurrent TB patients, 20 HCs)	16S rRNA sequencing (V3–V4 region); Illumina MiSeq platform; OTUs	Increase in α diversity	Decrease in *Prevotella*; increase in *Escherichia* and *Streptococcus*	Unknown	It affects immune status and correlates with patient prognosis and outcomes	[30]
2014	Mouse (*n* = 5/group, group = 4)	16S rRNA sequencing (V1‐V2 region); 454 FLX pyrosequencing platform; OTUs	Initial decrease in diversity followed by recovery	Decrease in *Lachnospiraceae* and *Ruminococcaceae*	Unknown	Cross‐talk between resident microbiota and mucosal immune system	[31]

Abbreviations: ASVs, amplicon sequence variants; GM, gut microbiota; HCs, healthy controls; ITS, internal transcribed spacer; KO, KEGG Orthology; LTBI, latent TB infection; OTUs, operational taxonomic units; PTB, pulmonary tuberculosis; Refs, references; SCFA, short‐chain fatty acid; TB, tuberculosis; TBM, tuberculous meningitis; TBW, TB patients after week treatment; TBZ, TB patients at day zero; *Year, the year the study was published.

### Reduced microbial diversity and TB progression

The GM of TB patients exhibits marked biodiversity loss compared to healthy individuals. Metagenomic sequencing of 46 TB patients and 31 controls revealed significant reductions in α‐diversity indices (species richness and abundance), with *Haemophilus parainfluenzae*, *Roseburia inulinivorans*, and *Roseburia hominis* depletion forming a diagnostic signature (AUC = 0.846) [27]. This finding is corroborated by longitudinal studies showing persistent GM structural changes posttreatment [34]. The significantly reduced GM diversity is not only directly related to the severity and progression of the disease, but also closely associated with the treatment effect. Clinical studies have further confirmed that patients with low GM diversity have an increased risk of treatment failure and a faster disease progression rate [35, 36]. Mechanistically, diminished diversity compromises gut barrier integrity, increasing permeability for MTB metabolites to trigger systemic inflammation [22]. The clinical relevance of diversity loss is further evidenced by reduced short‐chain fatty acids (SCFAs)‐producing genera (such as *Faecalibacterium*, *Roseburia*, *Eubacterium*, and *Phascolarctobacterium*). These genera are critical for maintaining immune homeostasis through butyrate‐mediated macrophage regulation [28], and their reduction impair macrophage bactericidal activity and antimicrobial peptide synthesis [37].

The GM of healthy individuals produces SCFAs and other metabolites that maintain the integrity of the gut epithelial barrier and promote immune tolerance [38]. In contrast, GM imbalance in TB patients, such as reducing *Lactobacilli* and *Bifidobacteria*, can impair gut barrier function [39]. This impairment increases gut permeability, allowing pathogens and inflammatory mediators to enter the bloodstream more easily and triggering systemic inflammation [22]. This “leaky gut” phenomenon can activate the innate immune system, increasing the production of inflammatory cytokines such as tumor necrosis factor‐α (TNF‐α) and interleukin‐6 (IL‐6), which inhibit macrophage antibacterial activity and enhance susceptibility to MTB [40, 41]. For example, Madeleine R. Wood et al. found that the increase in specific microbial communities in the gut of TB patients, such as *Prevotella* and *Veillonella*, are associated with disease progression and poor prognosis [42].

The systemic inflammatory response and metabolic changes triggered by MTB infection can also further exacerbate the loss of GM diversity through immune and neuroendocrine pathways, forming a vicious cycle. A large number of pro‐inflammatory cytokines released by the chronic inflammation caused by MTB infection, such as interferon‐γ (IFN‐γ) and IL‐17, can directly or indirectly affect the composition of GM, further reducing the types and quantities of microorganisms that have already decreased due to TB [32, 43]. These findings indicate that the GM ecosystem balance in TB patients is disrupted, potentially exerting profound effects on the host's immune function.

### Changes in GM composition and TB progression

MTB infection induces characteristic restructuring of GM at various taxonomic levels. *Proteobacteria* (particularly *Enterobacteriaceae*) and *Bacillota* show marked expansion, while *Bacteroidota* (especially *Prevotella*) and *Actinobacteria* (including *Bifidobacterium*) populations decline [16, 25, 28]. Parallel alterations are observed in fungal communities, with *Ascomycota* dominance replacing *Basidiomycota* [24]. At the genus level, the abundance of *Bacteroides* and *Prevotella* increases, whereas *Blautia* and *Bifidobacterium* decrease in TB patients [24]. In terms of specific species, *Phocaeicola dorei*, *Escherichia coli*, *Prevotella copri clade C*, and *Akkermansia muciniphila* are enriched in TB patients, while *Phocaeicola vulgatus*, *Alistipes putredinis*, *Prevotella copri clade B*, and *Prevotella SGB1589* are more abundant in healthy controls [16, 25, 28]. Additionally, in TB patients, the abundance of *Saccharomyces* increases while *Aspergillus* decreases [24]. Notably, *Escherichia* and *Streptococcus* enrichment correlates with impaired immune status, whereas *Lachnospiraceae* depletion is associated with disrupted gut barrier function [25, 30]. These compositional shifts may establish a pro‐inflammatory milieu through multiple pathways: (1) Reduced mucin‐degrading *Prevotella* compromises intestinal epithelial integrity; (2) *Enterobacteriaceae* overgrowth promotes lipopolysaccharide (LPS)‐mediated Toll‐like receptor (TLR) 4 activation; (3) *Bifidobacterium* deficiency attenuates regulatory T cell (Tregs) responses.

These changes in GM also have a reverse effect on the infection process of MTB. For example, the alterations in the intestinal microenvironment caused by the decrease of *Prevotella* and the excessive proliferation of *Enterobacteriaceae* make it easier for MTB to breach the intestinal barrier and enter the bloodstream, thereby exacerbating the systemic infection. Moreover, the systemic inflammation caused by the infection of MTB will continuously disrupt the composition of the GM. For instance, it will further inhibit the growth of beneficial bacteria such as *Bifidobacterium*, reinforcing this vicious cycle [2, 30, 44].

### Decrease in SCFA‐producing bacteria and mutualistic populations and TB progression

SCFA‐producing bacteria, including *Faecalibacterium*, *Roseburia*, and *Eubacterium*, are markedly reduced in TB patients, paralleled by *Bacteroidota* depletion and *Bacillota* expansion [28]. These metabolic shifts critically impact host immunity, as SCFAs (acetate, propionate, butyrate) typically enhance anti‐inflammatory responses in monocytes and macrophages through G protein‐coupled receptor activation [45]. Namasivayam et al. identified butyrate reduction as a key severity marker, with levels inversely correlating with cavitary lesion development [29]. Li et al. further elucidated that SCFA deficiency disrupts MTB clearance mechanisms by attenuating immune regulation pathways [45]. The collective depletion of SCFA producers creates a dysbiotic environment favoring MTB persistence and systemic inflammation. Collectively, these findings suggest that the decrease in SCFA‐producing bacteria may significantly affect TB progression.

The GM influences TB progression through immunometabolic crosstalk and systemic immune modulation. Segal and Lachmandas et al. identified SCFA‐mediated suppression of IFN‐γ and IL‐17A as a critical susceptibility mechanism, impairing T helper cell (Th)1/Th17 responses essential for MTB containment [46, 47]. Yu et al. highlighted gut–lung axis interactions, where *Bacillota* reduction and *Pseudomonadota* expansion alter pulmonary microbiota and immune dynamics [48]. Khan et al. further demonstrated that GM dysbiosis skews Th17 differentiation, compromising alveolar macrophage function and perpetuating MTB survival [49]. Mayer‐Barber's review synthesizes these mechanisms, emphasizing GM's role in regulating inflammatory cytokine cascades and granuloma formation [50]. Collectively, these insights underscore GM modulation as a strategic target for adjunctive TB therapies.

Commensal species such as *Lactobacillus* and *Bifidobacterium* are significantly diminished in TB patients, weakening gut defense mechanisms against MTB invasion [30]. Yang et al. revealed that *Bacteroides fragilis* enhances anti‐TB immunity via long noncoding RNA regulation, while broader mutualistic depletion compromises pathogen exclusion through reduced lactic acid and hydrogen peroxide production [51, 52]. This loss impairs dendritic cell antigen presentation and effector T cell activation, critical for adaptive immunity [53, 54]. Additionally, mutualistic bacteria reduction elevates luminal pH, facilitating pathogen colonization and systemic dissemination of MTB through compromised mucosal barriers [55].

In addition, during MTB infection, changes in the host's nutritional status and metabolic state, such as malabsorption and metabolic disorders, will significantly reduce the quantity and function of SCFA‐producing bacteria and symbiotic flora. The decrease in the quantity of these flora, in turn, weakens the intestine's ability to resist MTB, promoting the growth and spread of MTB, thus forming a relationship of mutual influence [56]. Thus, this reduction in probiotic populations may lead to impaired adaptive immune responses, exacerbating the progression of TB.

### Increase in pathogenic bacteria and TB progression

TB progression is associated with pathogenic bacterial expansion, including *Pseudomonadota* and *Actinomycetota* in sputum and fecal samples [30, 57]. Cheung et al. observed phylum‐level shifts favoring *Pseudomonadota/Bacteroidota* in TB patients versus *Bacillota* dominance in controls [57]. Pathobiont overgrowth exacerbates disease through toxin‐mediated gut epithelial damage, niche competition suppressing beneficial flora, and gut–lung axis interactions amplifying pulmonary inflammation [58, 59]. For instance, Luo et al. linked *Streptococcus* enrichment to recurrent TB, while Dumas et al. demonstrated GM modulation of pulmonary immunity via bone marrow‐derived immune cell reprogramming [30, 60]. These pathogens further disrupt immune homeostasis through LPS‐driven TLR4 activation and bile acid metabolism alteration [32, 44].

Moreover, the changes in the immune response triggered by MTB infection will also create a more favorable environment for the proliferation of pathogenic bacteria. For example, the disruption of immune homeostasis caused by the infection allows the pathogenic bacteria that were originally suppressed in the intestine to multiply in large numbers. The increase in the number of pathogenic bacteria, through various pathways such as toxin‐mediated damage to the intestinal epithelium and alteration of the function of immune cells, further promotes the infection of MTB and the progression of the disease [58, 59]. These findings reveal the close relationship between GM and TB progression, providing new insights for disease prognosis assessment and personalized treatment.

## THE INTERACTION BETWEEN GM AND ATD

The interplay between GM and TB treatment has emerged as a critical research focus, driven by its dual role in modulating host immunity and interacting with ATD. Emerging evidence indicates that GM not only responds to ATD exposure but also actively influences drug metabolism and treatment outcomes. Additionally, ATD‐induced GM alterations may contribute to adverse drug reactions. These insights highlight GM as a potential target for optimizing TB therapeutics through improved efficacy and reduced toxicity. This section systematically examines three key aspects of GM–TB interactions: (1) ATD‐induced GM dysbiosis, (2) GM‐mediated modulation of ATD pharmacokinetics/pharmacodynamics, and (3) the mechanism of interaction between GM and ATD (Table 2) [18, 29, 49, 61, 62, 63, 64, 65, 66, 67]. Elucidating these mechanisms could enable personalized treatment strategies to enhance clinical outcomes.

**TABLE 2:** Effects of anti‐TB treatment on GM.

Refs	Year	Drugs	Species (Sample size)	Analysis method	Changes in GM	Impact or significance
[61]	2024	HRZE	Human (*n* = 24, including 5 naive TB patients, 6 DS‐TB, 10 DR‐TB‐inj–, 3 DR‐TB‐inj+)	16S rRNA sequencing (V3–V4 region); Illumina platform; OTUs/ASVs	Reduced α diversity; decrease in *Collinsella*, *Bacillota*, and *Prevotella*; increase in *Lactobacillus* (*Bacillota*)	May influence TB progression and treatment outcomes
[62]	2023	INH	Mouse (*n* = 7/group, group = 10)	16S rRNA sequencing (V3–V4 region); Illumina NovaSeq platform; ASVs	Significant increase in α diversity; increase in *Muribaculaceae* and *Bifidobacterium*; decrease in *Bifidobacteria*	Impairment of the immune system, increased risk of liver damage
[63]	2023	HRZ	Mouse (*n* = 5–7/group, group = 4)	HPLC/MS‐MS	Rapid and significant changes in GM composition	Affects the pharmacokinetics of certain TB antibiotics
[18]	2021	INH	Human (*n* = 99, including 29 naive TB patients, 29 TB2MT, 19 TB6MT, 22 HCs)	16S rRNA sequencing (V3–V4 region); ITS2; Illumina HiSeq. 2500 platform; ASVs	Reduced diversity of bacteria and fungi, with altered composition; dysbiosis characterized by a decrease in beneficial bacterial and fungal overgrowth; increase in *Bacteroides*, decrease in *Actinomycetota* and *Pseudomonadota*	Increased susceptibility to TB and limitation in INH‐mediated MTB clearance
[64]	2020	INH	Mouse (*n* = 4–5/group, group = 6)	16S rRNA sequencing (V3–V4 region); Illumina MiSeq platform; ASVs	Decrease in beneficial symbiotic bacteria; increase in *Bifidobacterium* and *Enterococcus*; decrease in *Bacteroides, Campylobacter*, and *Lactobacillus*	Impairment of T cell activation, proliferation, and memory T cell formation, weakening the immune response to INH treatment
[65]	2019	HRZ	Human (*n* = 84, including 13 HCs, 10 latent TB, 28 active TB, 13 TB with 1‐week therapy, 10 TB with 2‐week therapy, 10 cured TB)	qRT‐PCR	Significant reduction in α diversity; decrease in *Bacillota*, increase in *Bacteroides*	Risk factors for TB progression and prognosis
[49]	2019	HZ/R	Mouse (*n* = 5/group, group = 5)	16S rRNA sequencing (V4 region); Illumina HiSeq. 2500 platform; OTUs	HZ: Increase in *Bacteroidota*; R: Decrease in *Bacillota*, increase in *Verrucomicrobia* and *Bacteroidota*	Changes in GM metabolites in peripheral circulation may affect alveolar macrophage metabolism, increasing MTB burden, and negatively influencing macrophage immunity against MTB
[29]	2017	HRZ	Mouse (*n* = 4–5/group, group = 11)	16S rRNA sequencing (V3–V4 region); Illumina MiSeq platform; ASVs	Significant reduction in diversity; decrease in *Bacillota*, increase in *Erysipeloclostridium*	Affects host resistance to disease and immune function
[66]	2017	HRZE	Human (*n* = 120, including 50 uninfected controls, 19 treatment, 25 LTBI controls, 26 cured controls)	16S rRNA sequencing (V3–V4 region); Illumina MiSeq platform; ASVs; Metagenomic sequencing; Hiseq. 4000; Whole metagenome data	Decrease in *Ruminococcus*, *Eubacterium*, *Lactobacillus*, and *Bacteroides*; increase in *Erysipeloclostridium* and *Prevotella*	Microbiota disturbance and variability in peripheral immunity may affect the efficacy of TB treatment
[67]	2016	Broad‐spectrum antibiotics	Mouse (*n* = 4–5/group, group = 5)	Cultivable microbes (plating on different media); qRT‐PCR for specific bacterial species; ASVs	Reduced diversity; increase in *Enterococcus*; decrease in *Bifidobacterium*, *Lactobacillus*, *Campylobacter*, and *Bacteroides*	It promotes the growth of MTB in the lungs and facilitates its spread to other organs.

Abbreviations: DR‐TB‐inj–, drug‐resistant tuberculosis treated without injectable agents; DR‐TB‐inj+, drug‐resistant tuberculosis treated with injectable agents; EMB, ethambutol; HPLC/MS‐MS, high‐performance liquid chromatography/tandem mass spectrometry; HRZE, Isoniazid (INH, H), Rifampicin (RIF, R), Pyrazinamide (PZA, Z), Ethambutol (EMB, E); MTB, *Mycobacterium tuberculosis*; qRT‐PCR, quantitative reverse transcription polymerase chain reaction; TB2MT, TB patients after 2 months of treatment; TB6MT, TB patients after 6 months of treatment.

### Impact of ATD on GM

As the therapeutic mainstay for TB, ATD exerts broad‐spectrum effects extending beyond MTB to disrupt host GM homeostasis. Key mechanisms include: (1) reduced microbial diversity, (2) depletion of beneficial taxa (e.g., SCFA‐producing bacteria), (3) promotion of antibiotic resistance, (4) gut–immune axis dysregulation, and (5) secondary complications [68, 69]. Wipperman et al. demonstrated that standard ATD regimens significantly diminish GM diversity, particularly affecting *Roseburia* and *Faecalibacterium* populations, which correlated with immune variability and potential treatment efficacy reduction [66]. Subsequent work by the same group linked ATD‐driven GM changes to heightened inflammatory responses in TB patients, underscoring the clinical relevance of microbial balance [70]. Longitudinal analyses by Namasivayam et al. revealed persistent dysbiosis patterns during TB treatment, characterized by decreased *Bacteroidota* and increased *Pseudomonadota* abundance, potentially compromising host immunity and disease resistance [29]. Importantly, Khan et al. identified functional consequences of this dysbiosis, showing that impaired butyrate production weakens anti‐MTB immune responses [49]. These collective findings position GM integrity as a critical determinant of TB treatment success.

### Influence of GM on ATD metabolism

The GM‐ATD relationship exhibits bidirectionality, with GM actively participating in drug absorption, distribution, metabolism, and excretion processes [71, 72, 73]. Clinical studies demonstrate that GM metabolic activity can modify drug bioavailability and toxicity profiles. Maji et al. identified specific bacterial taxa involved in rifampicin (RIF) metabolism, directly influencing drug absorption dynamics [28]. Negi et al. further established that GM disruption impairs isoniazid (INH)‐mediated MTB clearance while exacerbating tissue pathology, suggesting microbiome‐dependent treatment responses [64], Certain supportive therapeutic applications can reduce the toxic and side effects of ATD. For example, Li et al. demonstrated that *Lactobacillus casei* supplementation alleviates ATD‐induced gastrointestinal toxicity in murine models via SCFA regulation [45]. These findings emphasize the need for microbiome‐informed dosing strategies in TB management.

### The mechanism of interaction between GM and ATD

**FIGURE 3:**
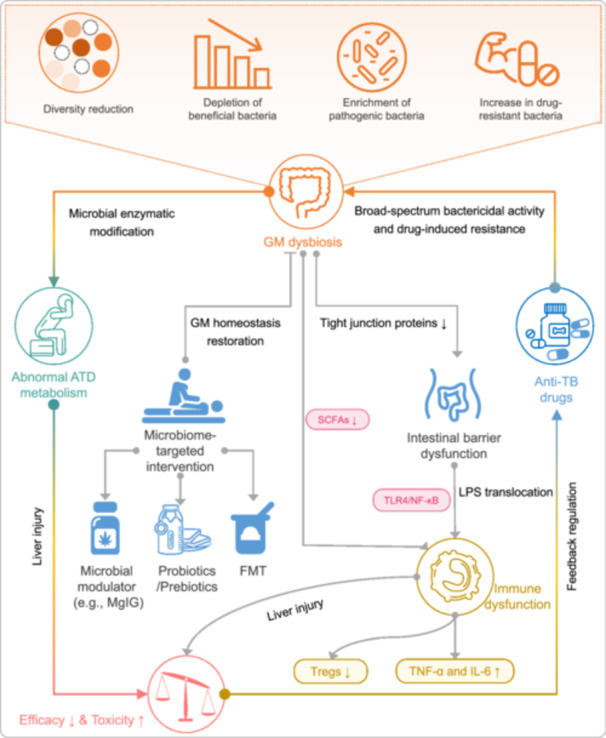
Bidirectional interactions between anti‐TB drugs (ATD) and gut microbiota (GM), and their impact on GM homeostasis. This figure illustrates: (1) The detrimental effects of ATD on GM compositions and functions, mediated through multiple mechanisms including the induction of intestinal inflammation, disruption of epithelial barrier integrity, metabolic perturbations, and direct bactericidal activity. (2) How GM dysbiosis reciprocally modulates the pharmacodynamics and toxicity of ATD, through mechanisms including altered drug metabolism, impaired immune modulation, reduced drug bioavailability, enhanced hepatotoxicity, and the facilitation of antimicrobial resistance. (3) Emerging therapeutic strategies aimed at restoring GM homeostasis, such as probiotics supplementation and administration of magnesium isoglycyrrhizinate, which may attenuate inflammation, promote barrier repair, and re‐establish microbial balance. Overall, this figure highlights the complex, bidirectional crosstalk between the GM and ATD, and suggests the potential of microbiota‐targeted interventions to optimize antituberculosis therapy. FMT, fecal microbiota transplantation. MgIG, magnesium isoglycyrrhizinate.

The interaction mechanism between GM and ATD is complex and diverse, involving physiological and biochemical processes at multiple levels. Next‐Generation Sequencing (NGS)‐driven pharmacomicrobiomics reveals GM's role in anti‐TB drug efficacy and toxicity. For example, microbial β‐glucuronidase activity reduces INH bioavailability through enzymatic inactivation, whereas RIF‐induced dysbiosis alters bile acid metabolism, increasing the risks of hepatotoxicity. Functional metagenomic analyses identify microbial pathways capable of detoxifying drug metabolites, suggesting potential targets for adjunctive therapies [40, 74, 75]. These findings align with Doestzada et al.'s proposal that GM can alter drug pharmacodynamics through both direct enzymatic modification and indirect immune or metabolic modulation, potentially explaining interindividual variability in ATD efficacy and tolerability [76]. This section highlights the multifaceted nature of GM–ATD interactions and sets the stage for a detailed examination of the specific mechanisms involved (Figure 3).

#### Mechanisms of ATD‐induced GM dysbiosis

Recent studies have elucidated the mechanisms by which ATD induces GM dysbiosis. For instance, Wu et al. [77] demonstrated that second‐line anti‐TB drugs used in the treatment of rifampicin‐resistant TB (RR‐TB) significantly alter the structural composition of the intestinal microbiota. Specifically, the relative abundance of beneficial species such as Prevotella copri decreases, while potentially pathogenic species like *Escherichia coli* and *Salmonella enterica* increase. This shift disrupts the gut's immune homeostasis and impairs the host's ability to mount an effective immune response against MTB. Functional analysis revealed that the biosyntheses of phenylalanine, tyrosine, and tryptophan are significantly inhibited during treatment, affecting the production of essential metabolites and neurotransmitters crucial for maintaining gut health and immune function.

Pei et al. [78] further explored the GM characteristics in TB patients experiencing liver injury following anti‐TB treatment. The study found that anti‐TB treatment leads to decreased microbial diversity and significant structural changes in the GM. At different time points, distinct differences in microbial composition were observed between patients with and without liver injury. These findings suggest that specific alterations in the GM may contribute to drug‐induced liver injury (DILI) in TB patients, potentially through modulating the host's immune and inflammatory responses.

#### Mechanisms of GM modulation of ATD pharmacokinetics/pharmacodynamics

New research has shed light on how GM influences ATD metabolism. Gong et al. [79] investigated the protective effect of magnesium isoglycyrrhizinate (MgIG) against anti‐TB DILI in mice. The study showed that MgIG significantly ameliorated HRZE‐induced liver injury by modifying the GM composition. Specifically, MgIG increased the abundance of beneficial bacteria such as Lactobacillus and enhanced the expression of tight junction proteins, improving intestinal barrier function and reducing intestinal permeability. This, in turn, decreased the levels of LPS and inhibited the activation of the LPS/TLRs/NF‐κB signaling pathway, which is associated with inflammation and liver injury. These findings highlight the role of GM in modulating the gut–liver axis and immune responses, thereby influencing the pharmacodynamics of ATD. Pei et al. [78] also showed that different anti‐TB drugs or drug combinations induce distinct GM profiles in mice models of liver injury. Each drug or combination causes dysbiosis characterized by unique changes in bacterial genera, which may affect the metabolism and toxicity of the drugs. These findings underscore the importance of considering specific microbiota signatures when evaluating the effects of different ATD on the host.

In summary, the mechanisms underlying the interaction between GM and ATD are multifaceted. ATD can induce dysbiosis through altering microbial composition and function, while GM can modulate ATD pharmacokinetics/pharmacodynamics by affecting drug metabolism and host immune responses. Understanding these mechanisms is essential for developing microbiome‐targeted interventions to optimize TB treatment efficacy and safety.

## GM AND TB IMMUNE RESPONSE

The host immune system serves as a critical defense mechanism against pathogenic invasion, functioning as the body's primary protective framework [80, 81, 82, 83]. Advances in metabolomics have highlighted the bidirectional interactions between GM and immune regulation, revealing GM's capacity to modulate host immune responses both locally and systemically—though precise mechanistic pathways remain incompletely characterized [84, 85]. These intricate GM–immune interactions undeniably influence TB pathogenesis and progression through multiple pathways. In addition, recent mechanistic studies have demonstrated that alterations in specific gut microbial taxa can directly affect the magnitude and quality of the immune response to MTB, thereby modifying disease progression [86].

**FIGURE 4:**
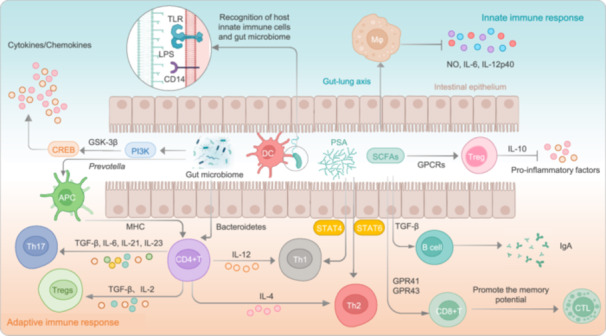
Multimodal regulation of host immunity by the gut microbiota (GM). The GM modulates the host immune system through multiple pathways. First, microbial components are recognized by pattern recognition receptors (e.g., TLRs) expressed on innate immune cells, activating downstream signaling cascades such as the phosphoinositide 3‐kinase (PI3K) pathway. This leads to the inactivation of glycogen synthase kinase 3β (GSK‐3β) and subsequent activation of cyclic adenosine monophosphate (cAMP) response element‐binding protein (CREB)‐dependent transcription of anti‐inflammatory genes, promoting the release of cytokines and other immunomodulatory factors. Second, microbial metabolites, such as SCFAs, bind to G protein‐coupled receptors (GPCRs) to drive the expansion of mucosal regulatory T cells (Tregs), thereby suppressing pro‐inflammatory responses. SCFAs also promote macrophage polarization through the gut–lung axis and enhance the memory function of CD8 + T cells, while stimulating B cell‐mediated secretion of immunoglobulin A (IgA), thereby strengthening mucosal and systemic immunity. In addition, specific bacteria taxa (e.g., *Bacteroides*, *Clostridium*, and *Prevotella*) promote the differentiation of CD4^+^ T cells into T helper 17 (Th17) and Tregs, contributing to the regulation of adaptive immunity. Moreover, bacterial polysaccharides can regulate T helper cell fate through activating signal transducer and activator of transcription 4 (STAT4) signaling in the presence of IL‐12 to promote Th1 differentiation, or STAT6 signaling under IL‐4 to promote Th2 responses, thus maintaining Th1/Th2 homeostasis.

Emerging evidence demonstrates three principal mechanisms through which GM modulates anti‐MTB immune responses: (1) direct immunocyte regulation, (2) metabolite‐mediated immune modulation, and (3) lung microenvironment alteration via the gut–lung axis. Elucidating these interactions provides critical insights into TB pathogenesis while informing novel preventive and therapeutic strategies. This section systematically examines GM‐immune interplay through four dimensions: innate immunity, adaptive immunity, gut–lung axis dynamics, and immunocyte functional regulation (Figure 4). Collectively, these advances offer transformative perspectives for TB management.

### GM and innate immune response

GM modulates innate immunity through multiple mechanisms, establishing a sophisticated regulatory network. Microbial components such as LPS and peptidoglycan, engage host pattern recognition receptors—including TLRs and NOD‐like receptors—expressed on macrophages, dendritic cells, and intestinal epithelia [87, 88]. Activation of pattern recognition receptors by microbe‐associated molecular patterns initiates downstream signaling cascades that regulate cytokine/chemokine production and inflammatory responses [89, 90]. Brown et al. [91] identified a TLR1/TLR2‐Dectin‐1‐mediated phosphoinositide 3‐kinase pathway activation, resulting in glycogen synthase kinase 3β inactivation and cyclic adenosine monophosphate (cAMP) response element‐binding protein‐dependent anti‐inflammatory gene expression—a critical frontline defense mechanism.

Beyond pathogen recognition, the GM regulates the innate immune response to TB through its metabolites such as SCFAs, including acetate, propionate, and butyrate. Depleted SCFA levels disrupt IL‐10 production and macrophage polarization, while increased LPS from pathobionts activate TLR4 signaling, amplifying pro‐inflammatory cascades. Secondary bile acid metabolism perturbations further contribute to macrophage dysfunction, illustrating the complex interplay between microbial metabolites and host immunity [92, 93].

GM further supports immune regulation through intestinal barrier maintenance [94]. By enhancing mucus production, tightening epithelial junctions, and inducing antimicrobial peptide secretion, GM creates physicochemical barrier against pathogen translocation [37, 95]. Barrier integrity prevents excessive inflammation and systemic immune activation, while GM dysbiosis disrupts this equilibrium—predisposing to inflammatory bowel disease, metabolic disorders, and autoimmunity [96]. These findings position GM as both a central innate immune regulator and potential therapeutic target.

Recent studies have also shown that GM can influence the function of alveolar macrophages, which are crucial for the initial defense against MTB in the lungs. For instance, Khan et al. [49] demonstrated that treatment with certain antituberculosis drugs, such as INH and pyrazinamide (PYZ), can alter the GM and subsequently impair the bactericidal activity of alveolar macrophages. This impairment is characterized by reduced expression of MHC II and decreased production of key anti‐mycobacterial cytokines like TNF‐α and IL‐1β, leading to increased susceptibility to MTB infection.

Furthermore, additional mechanistic insights reveal that GM‐derived metabolites can activate the NLRP3 inflammasome in alveolar macrophages, enhancing IL‐1β secretion, which is pivotal for early MTB control [21]. Moreover, studies have shown that GM‐derived butyrate not only modulates macrophage activity but also regulates gut mucus barrier repair via the macrophage/WNT/ERK signaling pathway, thereby indirectly influencing pulmonary immune defense against MTB [97].

### GM and adaptive immune response

Emerging evidence demonstrates that GM critically influences not only innate immunity but also orchestrates adaptive immune responses. The adaptive immune system, mediated by B and T lymphocytes, establishes antigen‐specific immunological memory for long‐term protection [98, 99]. GM profoundly modulates T‐cell differentiation patterns: *Bacteroides* and *Clostridia* species induce Treg development, *Bordetella pertussis* enhances Th1 cell maturation, while segmented filamentous bacteria, *Citrobacter rodentium*, and *Escherichia coli* drive Th17 differentiation in murine models [100, 101, 102]. Notably, Loftfield et al. [103] demonstrated in pulmonary studies that *Prevotella* species activate TLR2 signaling, stimulating antigen‐presenting cells to secrete IL‐1β, IL‐6, and IL‐23, thereby promoting Th17 differentiation from CD4^+^ T cells.

GM‐derived metabolites significantly regulate adaptive immunity. Butyrate, crucial for combating intracellular pathogens and malignancies, enhances CD8^+^ T cell metabolic fitness and memory potential [104]. SCFAs promote Tregs differentiation while suppressing pro‐inflammatory cytokine production, thereby maintaining immune homeostasis [105, 106, 107, 108]. Smith et al. [109] demonstrated free fatty acid receptor 2 dependent GM‐SCFA regulation of colonic Treg populations and colitis prevention in murine models. Concurrently, SCFAs modulate B cell metabolism and antibody production, with DA Peterson et al. [110] showing that SCFA‐mediated secretory IgA responses reinforce mucosal immunity against enteric pathogens. Furthermore, GM components like polysaccharide A from *Bacteroides fragilis* correct systemic T cell deficiencies and Th1/Th2 imbalances, as evidenced by comparative studies in germfree versus colonized animals [111]. These intricate interactions underscore the bidirectional GM‐immune crosstalk. However, GM dysbiosis disrupts this equilibrium, characterized by Treg depletion and pathogenic Th1/Th17 expansion, potentially driving chronic inflammation and autoimmunity [112]. These findings highlight GM's pivotal role in adaptive immune homeostasis and its therapeutic implications.

In the context of TB, GM dysbiosis has been shown to affect the balance of T‐cell subsets. For example, Segal et al. [46, 47] found that increased levels of SCFAs, such as butyrate, in human immunodeficiency virus (HIV) ‐infected individuals on antiretroviral therapy (ART) were associated with higher TB susceptibility. Butyrate inhibited the production of IFN‐γ and IL‐17A, which are crucial for controlling MTB infection, while promoting the induction of FoxP3+ Tregs that can suppress protective immune responses. Similarly, Lachmandas et al. [46, 47] demonstrated that butyrate could reduce pro‐inflammatory cytokine responses to MTB in human peripheral blood mononuclear cells (PBMCs) while increasing IL‐10 production, an effect that was independent of its HDAC inhibitory activity.

Moreover, emerging data indicate that shifts in the GM composition can influence antigen‐presenting cell function, thereby modulating Th1 and Th17 responses critical for MTB clearance. Specifically, experimental models have shown that GM dysbiosis leads to attenuated IFN‐γ production by T cells—a mechanism that may underpin increased TB susceptibility [53].

### GM and the gut–lung axis

The gut–lung axis, first conceptualized by Turner‐Warwick (1968) [113], represents a bidirectional immunoregulatory network connecting intestinal and pulmonary mucosal systems. Emerging evidence implicates this axis in respiratory infections, with Dumas et al. [60] proposing GM‐mediated modulation of MTB colonization through metabolite production, immune cell regulation, and pulmonary microenvironment remodeling.

GM influences pulmonary immunity through three principal mechanisms: (1) Circulating microbial metabolites (e.g., SCFAs) regulate alveolar macrophage function [60, 114]; (2) Bacterial components prime systemic immune responses; (3) Immune cell trafficking between mucosal sites. Conversely, pulmonary perturbations reciprocally affect gut homeostasis. Anti‐TB therapy exemplifies this interdependence—Wipperman et al. [70] observed that antimicrobial‐induced microbiome alterations during early TB treatment (≤14 days) may impair inflammatory resolution through systemic immune modulation. These findings elucidate the gut–lung axis's complex role in TB pathogenesis and therapeutic response.

Notably, GM perturbations by specific anti‐TB drugs may trigger cascading effects. Khan et al. [49] demonstrated that INH‐ and PYZ‐induced GM dysbiosis impairs alveolar macrophage functionality—a frontline defense against pulmonary MTB. Crucially, fecal microbiota transplantation (FMT) reverses this impairment, directly validating GM's role in maintaining alveolar macrophage homeostasis via the gut–lung axis. Emerging evidence further expands this paradigm: Gut‐derived microbial signals regulate lung dendritic cell migration and function, enhancing granuloma formation and anti‐MTB immunity [51, 52]. Conversely, high‐fat diet‐induced GM alterations remodel the pulmonary transcriptome via *NOS2* upregulation, compromising intracellular antimicrobial defenses and promoting MTB colonization [17]. Collectively, these findings delineate a multidimensional regulatory network through which GM influences TB pathogenesis and therapeutic outcomes via the gut–lung axis.

### GM and immune cell dynamics

GM critically regulates immune cell dynamics central to TB pathogenesis, particularly Th1‐polarized responses essential for macrophage activation via IFN‐γ signaling [83]. Mechanistic studies reveal GM's modulation of unconventional T cell subsets: Vorkas et al. [115] demonstrated GM‐mediated regulation of mucosal‐associated invariant T cells, while Constantinides et al. [116] identified γδ T cell functional modulation. Dysbiosis‐induced Th1/Th2 imbalance may compromise anti‐mycobacterial immunity [117].

GM further impacts systemic inflammation through intestinal barrier regulation. Dysbiosis increases intestinal permeability, permitting translocation of microbial products (e.g., LPS) that drives chronic inflammation—a potential accelerator of TB progression [58]. These collective mechanisms position GM as a critical modulator of TB immunopathology and a promising target for immunological interventions.

Recent mechanistic studies have also uncovered that GM can modulate the cytotoxic function of NK cells, enhancing the clearance of MTB‐infected cells. Furthermore, GM‐induced alterations in systemic cytokine profiles may shift the balance between pro‐inflammatory and regulatory immune responses, further influencing TB outcomes [118]. In addition, gut dysbiosis has been associated with increased intestinal permeability and upregulation of *NOS2* in lung tissues, which leads to reduced reactive oxygen species production and decreased expression of *DEFB1*, thereby creating a microenvironment favorable for MTB colonization [119].

## GM AS A POTENTIAL TARGET FOR DIAGNOSIS AND TREATMENT OF TB

**FIGURE 5:**
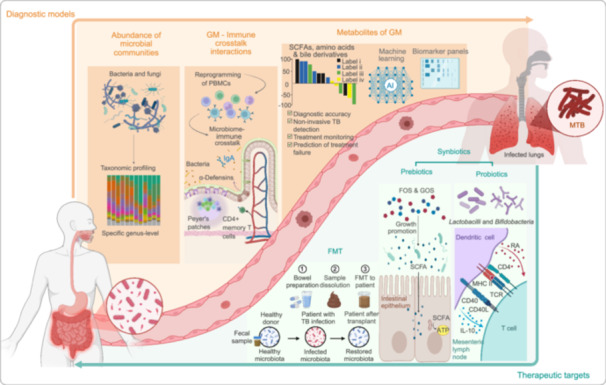
Gut microbiota (GM) as a diagnostic and therapeutic target in tuberculosis (TB). Emerging evidence highlights the GM as a key modulator of TB pathogenesis and a promising target for diagnosis and treatment. At the molecular level, the GM influences TB development through systemic circulation, with alterations in microbial composition, fungal–bacterial interactions, gut‐immune crosstalk, and metabolite profiles offering diagnostic potential. Elucidating these mechanisms is critical for advancing TB therapeutics. Microbiota‐targeted interventions, including probiotics, prebiotics, synbiotics, and FMT can restore GM homeostasis, reduce treatment‐associated complications, and improve gut and immune health.

The growing recognition of GM in human pathophysiology has positioned it as an emerging focus for TB management strategies. Mounting evidence demonstrates profound GM dysbiosis in TB patients, with these microbial alterations not only serving as disease biomarkers but potentially influencing clinical progression and therapeutic responses. This dual diagnostic‐therapeutic potential stems from GM's noninvasive accessibility and capacity to modulate both host immunity and xenobiotic metabolism. Current research explores two principal applications: (1) development of microbial signature‐based diagnostic tools, and (2) microbiome‐targeted interventions to enhance treatment efficacy. This section examines recent advances in GM‐based TB management approaches and their clinical implications (Figure 5).

### GM as a biomarker for the diagnosis and treatment monitoring of TB

#### GM as a diagnostic biomarker for TB

Emerging evidence highlights the capacity of GM alterations to serve as sensitive indicators of host health status, with particular relevance to TB diagnostics. Taxonomic profiling reveals characteristic patterns of reduced microbial diversity and specific genus‐level abundance changes in TB patients. Han et al. identified distinct bacterial‐fungal co‐occurrence networks that improved diagnostic accuracy when incorporated into combinatorial models [24]. Complementary work by Hu et al. demonstrated robust classification performance using bacterial abundance profiles alone, achieving effective discrimination between healthy controls (HCs) and TB patients [27]. Mechanistically, Namasivayam et al. established correlations between GM alterations and functional reprogramming of PBMCs, suggesting microbiome‐immune crosstalk could inform diagnostic development [34].

The diagnostic potential extends beyond taxonomic composition to microbial metabolic output. Metabolomic analyses reveal disease‐associated perturbations in SCFAs, bile acid derivatives, and amino acid profiles. Maji et al. specifically implicated depletion of SCFA‐producing taxa as a potential diagnostic marker, linking microbial metabolism to TB pathophysiology [28]. Compared to conventional diagnostics relying on sputum analysis (smear microscopy, culture) or tuberculin skin testing, GM‐based approaches offer distinct advantages in sample accessibility and analytical throughput. Lu et al. demonstrated this potential through machine learning (ML) integration of microbiome data, achieving diagnostic accuracy comparable to established methods while eliminating sputum collection requirements [120]. Luo et al. [121] developed fecal biomarker panels (e.g., deoxycholate, LysoPC 12:1) and a nomogram integrating age/sex to stratify latent TB infection (LTBI) progression risk. These developments position GM analysis as a promising platform for noninvasive TB detection and treatment monitoring.

#### Treatment monitoring based on GM metabolic characteristics

The research progress of GM metabolomics provides crucial evidence for treatment monitoring and individualized interventions. Sahu et al. [122] tracked partial GM restoration and metabolic normalization in TB patients during treatment. Luies et al. [123] identified GM‐derived metabolites (3,5‐dihydroxybenzoate, 3‐(4‐hydroxy‐3‐methoxyphenyl)propionate) predictive of treatment failure (AUC = 0.94). These findings lay the foundation for a TB efficacy evaluation system based on GM metabolic characteristics.

### GM as a therapeutic target for TB

TB therapies are known to disrupt GM equilibrium, impairing immunity‐associated metabolites like bile acids and SCFAs [34, 66, 124]. Emerging evidence supports GM modulation as a promising adjuvant approach for TB treatment [125, 126]. Accumulating evidence establishes GM's critical involvement in three therapeutic‐relevant processes: immunomodulation, drug biotransformation, and pathogen containment. This functional triad underpins growing interest in microbiome engineering as an adjunct to conventional anti‐TB regimens. Therapeutic strategies broadly fall into two categories: (1) pharmacological modulation of microbial communities, and (2) ecological restoration through microbiota transplantation.

#### Pharmacological modulation of microbial communities

In the research of TB treatment, pharmacological interventions primarily utilize probiotics, prebiotics, and synbiotics to reshape microbial communities. Diallo et al. conceptualized a “Host‐Microbiota‐Directed Therapy,” demonstrating improved treatment outcomes through microbiome optimization [127]. Experimental validation is provided by Gavrilova et al., who identified anti‐mycobacterial activity in *Lactobacillus* strains through direct microbial antagonism [128]. Negi et al. further elucidated an immunological mechanism, showing probiotic‐mediated restoration of histocompatibility complex class II expression on lung dendritic cells coupled with Treg cell reduction enhances mycobacterial clearance [53]. Although different studies consistently emphasize the importance of immunomodulation, suggesting a dominant therapeutic pathway, differences in implementation, effect evaluation, and dosing strategies have been observed. These variability issues underscore the need for further standardization of experimental protocols and a deeper exploration of optimal clinical strategies.

#### Ecological restoration through microbiota transplantation

Microbiota transplantation has emerged as a promising strategy for ecological restoration in TB therapeutics, aiming to rectify treatment‐associated dysbiosis by reconstituting balanced microbial communities. FMT, a therapeutic modality with well‐documented efficacy in *Clostridioides difficile* infection management, is now being explored for its potential in TB treatment optimization. Pioneering work by Trivedi et al. [129] demonstrated that FMT could effectively mitigate antibiotic‐induced GM dysbiosis during TB therapy, potentially enhancing treatment outcomes through microbiome stabilization. This finding is further supported by Eribo et al. [130], who reported that post‐antibiotic FMT administration not only accelerates microbial community recovery but also reduces treatment complications and may decrease transmission risks in drug‐resistant TB (DR‐TB) through microbiome‐mediated immune modulation.

Critical evaluation of existing evidence reveals that the therapeutic efficacy of FMT is modulated by three principal factors: donor‐recipient compatibility, temporal precision of intervention, and host‐specific biological variables. Rigorous donor screening protocols—particularly those incorporating microbial diversity metrics and pathogen exclusion criteria—have shown direct correlation with clinical success rates. The timing of FMT administration relative to antibiotic exposure emerges as a crucial determinant of microbial engraftment efficiency, with early‐phase interventions demonstrating superior ecological restoration capacity. Furthermore, interindividual variations in immune status, baseline GM architecture, and genetic predisposition significantly influence therapeutic responsiveness.

#### Integrated perspectives and future directions

Current research substantiates a paradigm shift toward integrated TB management frameworks that synergize conventional antimicrobial therapy with microbiome‐stabilizing interventions. Accumulating evidence from preclinical models and clinical observations suggests that GM modulation could address three persistent challenges in TB care: (1) treatment duration reduction through enhanced chemotherapeutic efficacy mediated by microbial β‐glucuronidase activity [53], (2) side effect mitigation via microbiome‐dependent optimization of drug metabolism pathways [62, 131], and (3) relapse prevention through sustained immunomodulation of tissue‐resident memory T cells [26, 132]. Notably, emerging combinatorial approaches demonstrate amplified therapeutic potential—controlled trials reveal that high‐fiber diets potentiate probiotic efficacy by elevating SCFA production, while polyphenol‐rich nutritional regimens suppress pro‐inflammatory taxa proliferation during anti‐TB treatment [133]. This metabolic‐immune crosstalk is further exemplified by Martineau et al.'s landmark discovery that vitamin D supplementation augments TB treatment responses through GM‐dependent induction of antimicrobial peptides [134].

The next frontier in TB therapeutics lies in rationally integrating pharmacological and ecological restoration strategies. To achieve this, future investigations must systematically decipher the tripartite interaction dynamics between gut ecosystems, pharmacological agents, and host immunity using longitudinal multi‐omics profiling (metagenomic, metabolomic, proteomic) coupled with gnotobiotic animal models. Concurrently, phase III clinical trials employing precision microbiota interventions should establish three critical parameters: (a) patient stratification biomarkers predictive of intervention responsiveness, (b) optimal therapeutic windows relative to antibiotic exposure phases, and (c) standardized efficacy metrics encompassing microbial diversity indices, inflammatory biomarkers, and treatment completion rates. The convergence of these research trajectories promises to advance personalized TB regimens tailored to individual microbial and immunological profiles. Such integrative approaches not only reinforce global TB control through improved treatment adherence and reduced transmission risks but also provide a mechanistic framework for developing next‐generation therapeutics targeting host–microbe–drug interactions.

## MULTI‐OMICS AND AI TECHNOLOGIES IN GM‐TB RESEARCH

### NGS: Revolutionizing genomic investigations

NGS, as a group of high‐throughput technologies, can rapidly and cost‐effectively analyze complete genomes, transcriptomes, and epigenomes. Unlike traditional Sanger sequencing, which processes individual DNA sequences sequentially, NGS achieves parallel processing of millions of DNA fragments through automated workflows. This breakthrough has transformed genomic research such as medicine and has been critical for pathogen characterization, drug resistance detection, and epidemiological surveillance [135]. Building on the technological developments, NGS has significantly advanced microbial community profiling.

#### Technologies, developments, and multifaceted applications

**FIGURE 6:**
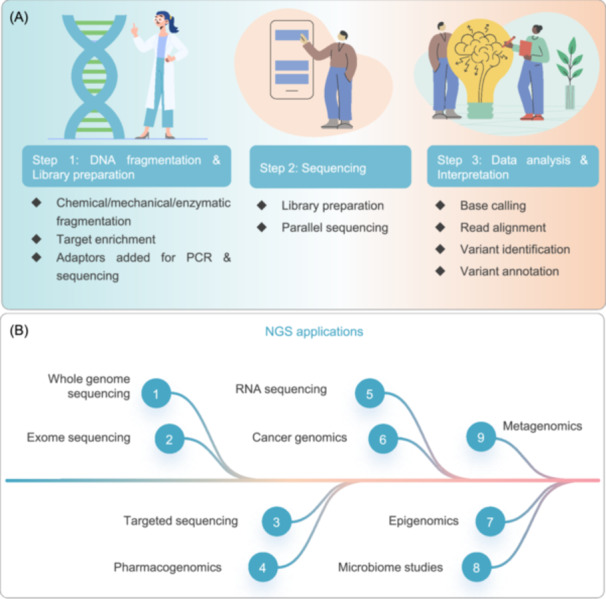
Overview of the next‐generation sequencing (NGS) workflow and its applications. (A) Schematic representation of the core stages in the NGS workflow: DNA fragmentation and library preparation, high‐throughput sequencing, and bioinformatics analysis and data interpretation. (B) Illustration of the diverse applications of NGS across genomics and biomedical research.

The core principle of NGS involves parallel sequencing of DNA or RNA fragments immobilized on solid surfaces such as beads or flow cells (Figure 6A). After a series of operations including adapter ligation and clonal amplification, sequencing clusters are generated. With diverse major detection methods, Illumina technology is widely applied, while Pacific Biosciences (PacBio) and Oxford Nanopore are proficient in long‐read sequencing [136].

The commercial development of NGS platforms began with the 454 pyrosequencer [137]. Early platforms differed in several aspects [138]. Subsequently, third‐generation sequencing platforms (such as PacBio and Oxford Nanopore), which can directly sequence single DNA molecules emerged (Table 3) [139, 140, 141, 142, 143, 144, 145, 146, 147, 148, 149, 150]. Emerging methods combine short‐read efficiency with long‐range genomic information.

**TABLE 3:** Comparison of the most important sequencing platforms of NGS.

Method	Properties	Refs
Illumina sequencing	Short‐read sequencing	[139, 140, 141]
	The most commonly used NGS platform	
	Relies on sequencing‐by‐synthesis technology (fluorescently labeled nucleotides are incorporated into a growing DNA strand during sequencing)	
	Identifying the detected resulting signal to determine the incorporated base	
	Simultaneously sequencing millions of short reads (typically 100–300 bp in length)	
	High accuracy,	
	Extensive bioinformatics support	
	Scalability	
	Wide range of applications, including whole‐genome sequencin, whole‐exome sequencing, targeted sequencing, and RNA‐ sequencin	
Ion torrent sequencing	Semiconductor Sequencing (detects the release of protons when a nucleotide is incorporated into the DNA strand)	[142, 143, 144]
	Eliminates the need for optical detection	
	Uses changes in pH to identify the base being added to the growing strand	
	Fast turnaround time	
	Lower cost	
	Shorter reads (ranging from 50 to 400 bp)	
	Commonly employed in targeted sequencing applications, such as gene panels and microbial sequencing, and for research requiring high‐speed results.	
PacBio sequencing	Long‐read sequencing	[145, 146, 147]
	Known as Single Molecule Real‐Time sequencing	
	Reading of much longer DNA fragments compared to Illumina and Ion Torrent platforms (average read lengths range from 10 kb to >100 kb)	
	Utilizes zero‐mode waveguide technology to capture real‐time sequencing events	
	Generating each read by combining DNA polymerase with nucleotides and detecting the light emitted by the combined bases	
	Advantageous for resolving complex genomic regions, structural variants, and repetitive sequences that are difficult to sequence with short‐read technologies	
	Used widely for de novo genome assembly, transcriptome analysis, and the study of large, complex genomes	
Oxford nanopore sequencing (Nanopore technology)	Based on the detection of changes in electrical current as DNA strands pass through nanopores embedded in a membrane	[140, 148, 149, 150]
	Real‐time sequencing method	
	Generating ultra‐long reads (with some individual reads extending over several megabases)	
	Offering a portable and flexible sequencing solution	
	It can be deployed in various environments using devices such as the MinION	
	Providing significant advantages in read length and real‐time sequencing, enabling applications such as rapid pathogen detection, environmental monitoring, and metagenomics	
	Higher error rate compared to Illumina and PacBio (but advances in software and base‐calling algorithms have steadily improved accuracy and reliability)	

Abbreviations: NGS, next‐generation sequencing; PacBio, Pacific Biosciences.

Different NGS methods are suitable for different experimental purposes. Illumina is the gold standard for large‐scale genomic analyses and PacBio and Oxford Nanopore are applicable to long‐read resolution, Ion Torrent and others have unique advantages in clinical applications. The emerging technologies such as single‐cell RNA‐sequencing (RNA‐Seq), have promoted NGS applications and facilitated the study of genetic diversity and cellular heterogeneity at unprecedented depth [151, 152].

The versatility of NGS covers multiple disciplines, and its core applications involve seven key areas (Figure 6B). In genomic research, whole‐genome sequencing (WGS) serves as the foundation, showing remarkable advantages in clinical applications [153]; targeted sequencing is a cost‐effective alternatives, and exome sequencing can identify pathogenic mutations [154]. NGS‐based RNA‐Seq has revolutionized transcriptome analysis [155, 156]. Epigenetics can analyze DNA methylation with the help of NGS [157, 158]. In microbial ecology, NGS has revolutionized metagenomics research [159, 160]. In oncology, NGS is used to identify genetic alterations, and liquid biopsy enhances its clinical impact [161]. Furthermore, it has been reported that pharmacogenomics optimizes therapeutic strategies through NGS to improve patient outcomes [162].

#### NGS‐driven microbial community profiling

NGS has fundamentally transformed microbial community analysis by overcoming the limitations of traditional culture‐dependent methods. Conventional approaches fail to detect approximately 80% of environmental microbes due to unculturability, whereas NGS enables comprehensive detection of microbial constituents through direct nucleic acid sequencing, providing insights into community composition, functional potential, and metabolic activity across diverse ecosystems [163].

For microbial profiling, distinct NGS strategies are used based on research goals. 16S rRNA gene sequencing is the gold standard for taxonomic classification at genus or species level. Whole‐genome shotgun sequencing analyzes functional genes and metabolic pathways, and metatranscriptomics captures community gene expression. Platform selection among Illumina, PacBio, and Nanopore technologies depends on read length, throughput, and error tolerance [135].

Deep sequencing enables high‐resolution diversity analysis, revealing dominant and low‐abundance microbial members. This helps identify keystone species, detect pathogens, and monitor population shifts. The depth of sequencing directly correlates with the accuracy of microbial interaction network reconstruction for precise modeling of community dynamics [164].

In clinical microbiology, NGS‐based profiling has elucidated the human microbiome's role in health and disease, such as dysbiosis in gut microbial communities linked to disorders. In environmental applications, NGS clarifies microbial contributions to biogeochemical cycles, and in extreme environment studies, it helps catalog extremophile communities and their adaptations [165].

#### Applications in GM‐TB research

**FIGURE 7:**
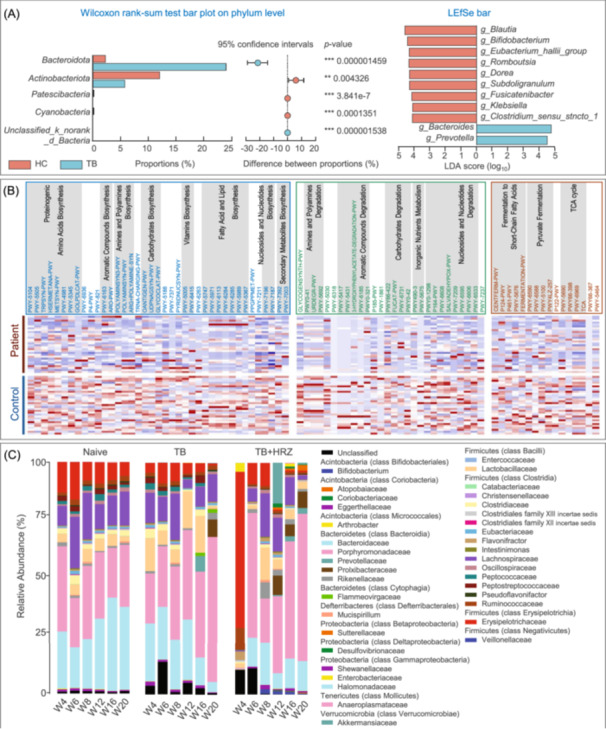
Applications of NGS in gut microbiota‐tuberculosis (TB) research. (A) Microbial composition analysis: Relative abundances of intestinal bacterial phyla in TB patients versus healthy controls (HC) were compared using the Wilcoxon rank‐sum test. Differentially abundant taxa were identified by linear discriminant analysis effect size (LEfSe) with LDA score > 4 and *p* < 0.05. Adapted with permission from [24], Copyright 2024, BMC. (B) Metabolic pathway analysis: Heatmap shows differential metabolic pathway abundance between TB and HC groups based on NGS‐derived functional profiling. Pathways were ranked according to consensus functional classification; statistical significance was determined using the Wilcoxon rank‐sum test with false discovery rate (FDR) < 0.1. Abundances were standardized as row Z‐scores. Adapted with permission from [27], Copyright 2019, Frontiers. (C) Longitudinal microbiota dynamics during anti‐TB therapy: Temporal changes in the average relative abundance of bacterial families were tracked using stool sample sequencing data across multiple treatment time points and experimental groups. Bacterial families are grouped by phylum and class in the accompanying color key. Time points are indicated along the *x*‐axis, grouped by treatment conditions. Adapted with permission from [29], Copyright 2017, BMC.

The GM interplay with MTB infection has emerged as a critical research frontier, particularly through the gut–lung axis concept. NGS, as the preeminent technology, plays a crucial role in GM‐TB research. This bidirectional communication pathway suggests GM modulation of pulmonary immunity, and NGS serves as the primary tool for characterizing microbial shifts during TB progression (Figure 7). High‐resolution sequencing reveals specific dysbiosis patterns in TB patients, including enrichment of pro‐inflammatory taxa and depletion of beneficial SCFA‐producing bacteria [11, 20].

For example, through 16S rRNA sequencing and shotgun metagenomics (both NGS‐based techniques), *Pseudomonadota* and *Bacillota* are consistently identified as dominant phyla in TB‐associated dysbiosis. NGS‐derived data also indicate that these microbial shifts correlate with heightened systemic inflammation and impaired granuloma stability, exacerbating lung pathology. Concurrent reductions in *Faecalibacterium* and *Bifidobacterium* populations, detected by NGS, diminish SCFA production, weakening gut barrier integrity and regulatory T‐cell differentiation—key factors in TB immune regulation [166, 167, 168].

Molecular mechanisms connecting GM alterations to TB pathology, which are deciphered with the help of NGS, involve metabolite‐mediated immune modulation. Host‐microbiota immune crosstalk studies demonstrate GM's systemic impact on TB pathogenesis. Increased intestinal permeability (“leaky gut”) allows microbial product translocation into circulation, triggering inflammatory pathways that compromise MTB containment. Dendritic cell‐mediated T‐cell priming in mesenteric lymph nodes and subsequent lung homing highlight the gut–lung immunological axis, with dysbiosis impairing this protective mechanism [39, 169].

Bioinformatics advancements, in dealing with the complexity of NGS data in GM‐TB studies, are essential. Tools like QIIME2 and MetaPhlAn2 enable robust taxonomic profiling, while PICRUSt and HUMANN2 predict functional potentials. ML integration identifies microbial biomarkers predictive of treatment response, facilitating personalized therapeutic strategies through multi‐omics data fusion [170, 171].

Emerging microbiome‐targeted therapies, guided by NGS‐revealed microbial profiles, extend beyond conventional probiotics. Postbiotics containing microbial‐derived SCFAs or antimicrobial peptides offer safer alternatives for immunocompromised patients. Engineered probiotics designed for targeted metabolite delivery and bile acid modulation represent a novel therapeutic frontier. For instance, genetically modified *Lactobacillus* strains engineered to constitutively express butyrate synthesis pathways have shown promise in preclinical TB models for restoring gut barrier function and anti‐inflammatory responses. Similarly, *E. coli Nissle 1917* derivatives equipped with bile salt hydrolase genes demonstrate enhanced capacity to mitigate RIF‐induced hepatotoxicity through bile acid detoxification [172].

FMT, an approach under investigation, also benefits from NGS. Early‐phase trials in TB patients, with NGS‐monitored microbial diversity recovery, explore FMT's potential to accelerate microbial diversity recovery post‐antibiotic treatment. Preliminary evidence suggesting reduced inflammatory markers and improved anti‐TB drug tolerance. However, safety protocols require rigorous standardization given the risk of pathogen transmission in immunocompromised hosts, which is also monitored using NGS [173].

The integration of microbiome science into TB management, facilitated by NGS‐derived microbial signatures, is progressing through two parallel pathways: predictive diagnostics and adjunctive therapies. Microbial signature panels derived from NGS data show potential as prognostic biomarkers, identifying patients at high risk of treatment failure or relapse based on baseline dysbiosis patterns. This stratification, enabled by NGS, allows personalized therapeutic regimens combining standard anti‐TB drugs with microbiome‐modulating interventions [174].

Longitudinal NGS monitoring reveals dynamic GM changes throughout anti‐TB therapy, highlighting critical intervention windows. The initial treatment phase, monitored by NGS, induces the most severe dysbiosis, suggesting early supplementation with SCFA‐producing strains or prebiotic fibers may preserve microbial resilience. Posttreatment phases, also tracked by NGS, exhibit incomplete GM recovery, warranting extended microbiota support strategies to prevent reinfection [175].

Future clinical trials, with a focus on NGS‐guided microbiome‐targeted therapy, must address key challenges in microbiome‐targeted therapy: (1) Standardization of intervention protocols across diverse populations, informed by NGS‐based population—specific microbial profiles; (2) Resolution of donor‐recipient compatibility issues in FMT, aided by NGS‐based microbial matching; (3) Development of TB‐specific microbial consortia with defined therapeutic functions, based on NGS‐predicted functional potentials; (4) Integration of host genetic factors influencing microbiome–drug interactions, explored through NGS‐enabled host–microbiome–drug interaction studies [176].

The ultimate goal involves developing microbiome‐informed precision medicine frameworks for TB, relying on NGS‐derived comprehensive microbial and host information. By correlating individual microbial profiles, obtained by NGS, with drug metabolism rates and immune response patterns, clinicians could optimize: (1) Antibiotic dosing schedules to minimize GM disruption, guided by NGS‐monitored drug–microbe interactions; (2) Probiotic/postbiotic selection based on strain‐specific functional deficits, identified by NGS‐based functional profiling; (3) Dietary recommendations tailored to microbial metabolic needs, deduced from NGS‐revealed microbial metabolic pathways [177].

NGS technologies have fundamentally redefined the GM‐TB research paradigm. From characterizing dysbiosis patterns to decoding metabolite–immune interactions, NGS‐driven insights are catalyzing a therapeutic revolution. The next decade will likely witness the clinical implementation of microbiome‐adjuvant therapies, transforming TB management from empirical antibiotic regimens to precision ecology‐based medicine, all centered around the application of NGS in GM‐TB research [178].

### Proteomics and the GM

#### Proteomic techniques for microbiome analysis

Proteomics, a crucial method for characterizing protein profiles in complex biological systems [179], can precisely identify and quantify proteins, detect posttranslational modifications (PTMs), and is useful for GM functional studies [180]. Dysbiosis in GM leads to changes in the gut metaproteome, related to conditions like metabolic syndrome and nutritional disorders [181].

Metaproteomics, which analyzes complete protein complements within environmental microbial communities [182], differs from conventional proteomics as it examines polymicrobial systems and captures organism‐specific PTMs that mediate microbial adaptation through mechanisms such as phosphorylation, acetylation, and oxidation [183]. Current analytical workflows combine advanced mass spectrometry (MS) with bioinformatics, while new synthetic databases improve peptide identification [183, 184].

However, there are challenges in computational requirements, standardization of protein grouping, and database construction. Metaproteomic analysis of neonatal fecal samples shows its potential in clinical diagnostics [184]. In GM analysis, MS‐based shotgun metaproteomics is commonly used with various sample sources (Table 4) [185, 186, 187, 188, 189, 190, 191, 192, 193, 194, 195, 196, 197, 198, 199], and has identified novel therapeutic targets in TB. Future developments should prioritize: (1) Diagnostic protocol optimization for clinical fecal sample processing, (2) Computational infrastructure enhancement for large‐scale data analysis, and (3) Integrative multi‐omics frameworks combining metaproteomic data with metabolomic and transcriptomic datasets to decipher host–microbe crosstalk.

**TABLE 4:** Comprehensive overview of proteomic strategies for identifying drug targets in TB treatment.

Mechanism	Advantage	Disadvantage	Refs
Identifying essential proteins in MTB via shotgun proteomics	It helps uncover critical pathways unique to the bacteria for selective drug targeting	Requires large protein databases; prone to sample complexity issues	[185]
Quantitative proteomics using SILAC or TMT	Enables precise comparison of protein expression under drug‐treated versus untreated conditions	Expensive; dependent on high‐quality MS facilities	[186]
Surface proteomics for membrane protein identification	Identifies proteins involved in host–pathogen interactions; potential vaccine candidates	Difficult to isolate membrane proteins; low abundance of targets	[187, 188]
PTM analysis of bacterial proteins	Reveals functional regulation mechanisms critical for survival or resistance	Complex data analysis, limited by incomplete PTM databases	[189]
Host proteomics to study immune responses	Identifies host pathways affected by TB, enabling host‐directed therapy discovery	May yield indirect targets; potential off‐target effects in therapies	[190]
Drug‐binding protein identification using affinity‐based approaches	Identifies bacterial proteins that directly interact with existing or experimental drugs	Limited to drugs with known mechanisms; may miss noncovalent interactions	[191]
Phosphoproteomics to map bacterial signaling pathways	Identifies kinases and phosphatases critical for TB survival and replication	It requires enrichment techniques and often suffers from low phosphopeptide abundance.	[192]
Comparative proteomics across drug‐resistant and susceptible strains	Identifies resistance‐related proteins and mechanisms	Interpretation can be complex due to strain‐specific variations	[193]
Proteomics for secretome analysis	Identifies proteins secreted by TB during infection, which are potential vaccine or drug targets	May miss low‐abundance proteins; secretome changes under different conditions	[194]
Integrative proteomics with transcriptomics	Correlates protein expression with transcriptional changes for robust target discovery	Proteomic data may not always correlate directly with transcriptomic data	[195]
Multi‐drug proteomic profiling	Examines protein changes under treatment with multiple drugs to detect synergistic effects	It may require extensive bioinformatics to interpret complex datasets	[196]
Functional proteomics with CRISPR screens	Combines proteomics with CRISPR gene editing to validate essential TB proteins as drug targets	High complexity; limited to labs with CRISPR expertise	[197]
Time‐resolved proteomics during TB infection	Tracks protein expression over infection stages, identifying targets for specific disease phases	Requires longitudinal sample collection; high costs	[198]
Proteomics‐guided vaccine target discovery	Identifies immunogenic proteins for designing TB vaccines	Proteins identified may lack sufficient antigenicity in human trials	[199]

Abbreviations: CRISPR, clustered regularly interspaced short palindromic repeats; MS, mass spectrometry; PTMs, posttranslational modifications; SILAC, stable isotope labeling by amino acids in cell culture; TB, tuberculosis; TMT, tandem mass tags.

#### Proteomic techniques in TB research

##### Shotgun proteomics

Proteomic techniques play crucial roles in TB research. Shotgun proteomics, using MS‐based workflows, enzymatically digests protein samples, separates peptides via multidimensional liquid chromatography (LC), conducts tandem MS analysis, and matches with databases for protein identification [200]. It can analyze complex peptide mixtures and is used for both qualitative and quantitative analyses.

In MTB research, shotgun proteomics has led to important discoveries. Nikitushkin et al. [201] performed LC‐MS proteomic profiling of latent MTB cells, identifying 1379 conserved proteins with 468 exhibiting significant quantitative shifts. Their work revealed metabolic adaptations involving methyl citrate cycle activation, glyoxylate shunt utilization, and pH‐dependent PhoP regulation. It also identified dormancy‐associated biotransformation enzymes critical for prodrug activation, which provides key insights for developing diagnostics and therapies against latent TB. Concurrently, Tucci et al. [202] analyzed MTB culture filtrates via LC‐MS/MS, detecting 1314 proteins including 6‐kDa eary secretory antigenic target secretion system X (ESX) secretion system components and pro‐glu/pro‐pro‐glu family (PE/PPE) virulence factors. Their identification of 46 O‐glycosylated proteins (33 novel) advanced understanding of MTB's antigenic landscape and survival strategies.

Clinical studies further validate shotgun proteomics' utility. Mateos et al. [203] identified distinct proteomic signatures in TB patients' biofluids, contrasting upregulated inflammatory mediators with enhanced innate immunity markers in uninfected contacts. From a therapeutic perspective, Villela et al. [204] confirmed guanosine monophosphate synthetase as essential through targeted validation, demonstrating that *guaA* deletion induces MTB lethality reversible via *Mycobacterium smegmatis* homolog complementation—establishing guanosine monophosphate synthetase inhibition as a viable therapeutic strategy.

##### Targeted proteomics

Targeted proteomics has emerged as a gold standard for precise protein quantification. It focuses on predefined protein subsets, different from untargeted methods that indiscriminately survey thousands of proteins [205]. There are three main methodologies in this field: Selected/multiple reaction monitoring (SRM/MRM), parallel reaction monitoring (PRM), and data‐independent acquisition (such as SWATH‐MS). SRM/MRM, implemented on triple quadrupole instruments, achieves exceptional selectivity via dual mass filtering and extended dwell times, enhancing sensitivity 10–100 × over full‐scan methods and matching immunoassay precision [206, 207]. PRM, while retaining SRM's precursor isolation, uses high‐resolution Orbitrap/time‐of‐flight (TOF) analyzers to capture full MS/MS spectra for better data analysis [208]. Data‐independent acquisition systematically partitions precursor windows across chromatographic elution profiles to generate comprehensive MS/MS maps [209].

Clinical applications demonstrate targeted proteomics' diagnostic potential. Yao et al. [210] identified a 9‐protein plasma signature distinguishing active TB from controls via analysis of 92 immune proteins, achieving 93.1% specificity for severe cases through ML. Robak et al. [211] developed a pleural effusion classification assay with 89% accuracy, differentiating TB‐associated pleural effusion from malignant/infectious etiologies. For drug resistance detection, Chen et al. [212] established a diagnostic panel (sCD14, PGLYRP2, FGA) demonstrating 92% sensitivity and 88% specificity for multidrug‐resistant TB (MDR‐TB) identification.

##### Single‐cell proteomics (SCP)

SCP has revolutionized cellular heterogeneity studies by resolving protein‐level variations that are not obscured in bulk analyses. Although single‐cell transcriptomics are popular in cellular resolution research, recent SCP advancements now enable functional proteome characterization at ultralow protein abundances (10⁻¹⁸–10⁻¹⁵ g/cell) [213]. Unlike bulk proteomics, averaging thousands of cells, SCP preserves cellular individuality. It can uncover rare pathogenic subpopulations, stochastic protein expression patterns, and dynamic PTMs in living systems [214]. This unique capability allows real‐time monitoring of protein dynamics in viable cells, contrasting with static snapshots from lysed cell aggregates (Table 5) [203, 210, 214, 215, 216, 217, 218, 219, 220, 221].

**TABLE 5:** Summarized proteomics techniques in microbiome and TB research.

Sr. no	Technique	Limitations/challenges	Potential solutions	Application in TB research	Mechanism of action	Refs
1	Shotgun metaproteomics	1. Complexity due to microbiome diversity 2. Difficulty identifying low‐abundance proteins 3. Database dependency	1. Enrich low‐abundance proteins via fractionation 2. Expand/update microbial protein databases 3. Improve computational algorithms	Profiling MTB proteins for biomarker discovery and therapeutic targets	Enzymatic digestion of proteins into peptides followed by LC‐MS/MS for identification and quantification	[203, 215, 216]
2	Targeted proteomics	1. Requires prior knowledge of target proteins 2. Limited ability to discover novel proteins 3. Quantitative biases	1. Combine with untargeted approaches for novel discoveries 2. Develop sensitive and robust quantitation methods	Quantitative measurement of MTB proteins for biomarker validation and drug target assessment	SRM/PRM techniques for selective peptide detection and quantification with high sensitivity	[210, 217]
3	SRM and PRM	1. Limited multiplexing capability 2. Requires high technical expertise 3. Expensive instrumentation	1. Develop cost‐effective automated platforms 2. Increase multiplexing capabilities 3. Provide user‐friendly software	Quantifying specific MTB proteins for validating biomarkers and drug targets	SRM/PRM in MS for selective peptide detection with high specificity	[218]
4	SCP	1. Low protein abundance 2. High variability and noise 3. Challenging sample preparation	1. Enhance sensitivity using nanotechnology‐based assays 2. Optimize single‐cell isolation 3. Use AI for noise reduction	Investigating protein expression at the single‐cell level to understand host immune responses to MTB	MS for analyzing proteins from individual cells, examining pathogen–host interactions	[219, 220]
5	Top‐down proteomics	1. Difficulty analyzing intact proteins 2. Low throughput compared to bottom‐up methods 3. Limited sequence coverage	1. Improve high‐resolution MS instrumentation 2. Combine top‐down and bottom‐up methods 3. Enhance workflow robustness	Characterizing intact MTB proteins, including posttranslational modifications	Direct analysis of whole proteins without prior digestion, identifying isoforms and modifications	[221]

Abbreviations: AI, artificial intelligence; LC‐MS, liquid chromatography‐mass spectrometry; PRM, parallel reaction monitoring; SCP, single‐cell proteomics; SRM/MRM, selected/multiple reaction monitoring; TB, tuberculosis.

In TB research, SCP has provided important insights. Chung et al. [222] revealed MTB remodels bone marrow mesenchymal stem cell proteomes to evade immune detection by downregulating antigen presentation machinery. Kaur et al. [223] demonstrated virulent MTB strains manipulate host differentiation pathways (Notch/Wnt), mitochondrial metabolism (oxidative phosphorylation suppression), and RNA splicing to establish persistent infection niches. At the host‐pathogen interface, Devasundaram et al. [224] identified divergent T‐cell responses to early secretory antigen‐6 (ESAT‐6) and Lpd antigens between latent and active TB patients via single‐cell cytokine profiling, suggesting pathogen‐directed immune modulation. These findings align with MTB's hypoxia adaptation mechanisms, where Forrellad et al. [225] observed upregulated DosR regulon proteins promoting dormancy in low‐oxygen bone marrow microenvironments.

##### Host–microbe interactions and proteomics in TB

MTB is a formidable pathogen due to its sophisticated co‐evolutionary adaptations [225]. The interplay between MTB and host immunity involves multilayered interactions spanning immune signaling, metabolic reprogramming, and cellular stress regulation [226], affecting host‐microbiome equilibrium and extracellular communication [227].

MTB actively subverts host defenses through molecular effectors such as Hip1 and ESAT‐6, disrupting T‐cell responses and creating an environment with restricted MTB spread and chronic inflammation [227]. Microbiome‐derived metabolites influence the host‐MTB metabolic crosstalk. For example, indole‐3‐propionic acid can inhibit MTB growth, showing its dual role in TB control [130].

**FIGURE 8:**
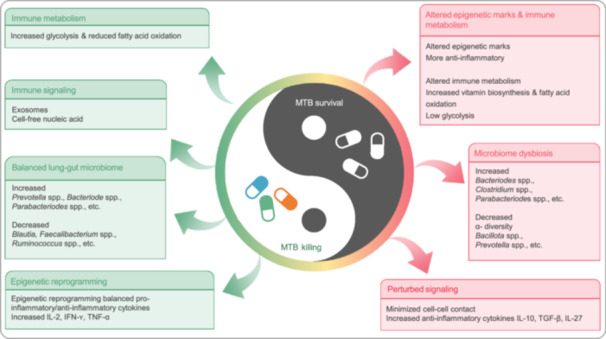
An overview of the host–microbe interactions in TB progression. The bidirectional influence of the lung–GM axis on host immunity and MTB survival. On the left (green), a balanced microbiome supports effective immune surveillance through enhanced immune signaling, a metabolically favorable state (characterized by elevated glycolysis and reduced oxidative phosphorylation), maintenance of stable immune epigenetic landscapes, and tightly regulated cytokines production, promoting MTB clearance. In contrast, the right side (pink) depicts a dysbiotic microbiome that contributes to impaired host defense through disrupted immune signaling (e.g., reduced cell–cell communication), metabolic dysregulation, and skewed pro‐/anti‐inflammatory responses, thus supporting MTB persistence and immune evasion. GM, gut microbiota; TB, tuberculosis.

Antibiotic therapies (e.g., INH, pyrazinamide [PZA]) induce GM dysbiosis in TB models, reducing macrophage microbicidal activity [130]. Proteomic techniques help study these interactions. Metaproteomics can analyze protein complements in the host‐MTB microbial communities, identifying proteins and PTMs for understanding molecular‐level interactions. Integrating proteomic data with other omics, as in future metaproteomics development, aids in understanding host–microbe crosstalk. Figure 8 synthesizes these integrated host mechanisms governing TB pathogenesis and resolution, guiding interpretation of proteomic data.

#### Potential for identifying novel therapeutic targets

Current anti‐TB regimens aim to achieve three primary objectives: complete MTB eradication, symptomatic relief, and prevention of disease transmission. The World Health Organization (WHO)‐endorsed directly observed treatment, short‐course strategy for drug‐sensitive TB combines four first‐line drugs during the intensive phase (INH, RIF, ethambutol [EMB], PZA) followed by a 4‐month continuation phase with INH and RIF. This standardized approach has contributed to the successful treatment of 66 million TB cases since 2000, as reported in the 2023 WHO guidelines [228, 229]. However, the management of MDR‐TB and extensively drug‐resistant TB (XDR‐TB) remains clinically challenging, requiring prolonged regimens (up to 2 years) with toxic second‐/third‐line agents that yield suboptimal outcomes.

The emergence of drug resistance stems from three principal mechanisms: (1) mutations in drug‐targeted genes or activating enzymes, (2) compensatory evolutionary adaptations, and (3) overexpression of efflux pump systems. These resistance pathways, coupled with the limited diversity of existing anti‐TB drugs, underscore the critical need for novel therapeutic strategies. Priority areas include developing agents capable of circumventing resistance mechanisms, shortening MDR‐TB treatment durations, and enhancing treatment tolerability.

Proteomics‐driven approaches are accelerating the discovery of next‐generation targets through two complementary strategies: (1) systematic identification of MTB vulnerabilities via essential protein mapping, and (2) characterization of host‐microbiome‐derived proteins with therapeutic potential. Table 6 summarizes proteomic methodologies for TB drug target identification [230, 231, 232, 233, 234, 235, 236, 237, 238, 239, 240, 241, 242, 243, 244, 245, 246, 247, 248, 249, 250], while Table 7 catalogs microbiome‐associated proteins demonstrating anti‐mycobacterial activity [251, 252, 253, 254, 255, 256, 257, 258, 259, 260]. These emerging resources provide a framework for rational drug design aimed at overcoming current therapeutic limitations in TB management.

**TABLE 6:** Discoveries using proteomics to identify drug targets in TB treatment.

Sr. no	Discovery/protein target	Role in TB pathogenesis	Methodology used	Refs
1	DnaK chaperone	Assists in protein folding under stress conditions, crucial for bacterial survival within host macrophages.	LC‐MS/MS‐based proteomics	[230]
2	Isocitrate lyase	Essential for MTB's glyoxylate shunt, enabling survival in nutrient‐limited conditions.	2D gel electrophoresis with MS analysis	[231]
3	ESX secretion system proteins	Involved in virulence and immune evasion by facilitating the secretion of effector proteins like ESAT‐6.	Label‐free quantitative proteomics	[232]
4	Mycothiol biosynthesis enzymes	Protects MTB from oxidative stress and maintains redox balance.	Targeted proteomics (MRM)	[233]
5	ATP synthase subunits	The key enzyme for energy production in MTB that inhibition can lead to bacterial death.	Top‐down proteomics	[234]
6	Phosphopantetheinyl transferase	Involved in lipid metabolism and mycolic acid synthesis, crucial for MTB cell wall integrity.	Shotgun proteomics	[235]
7	Clp protease complex	Degrades damaged or misfolded proteins, essential for bacterial stress response and survival.	LC‐MS/MS and bioinformatics	[236]
8	LprG‐Rv1410c lipid transport system	Facilitates lipid transfer for immune modulation, contributing to MTB's persistence in host tissues.	Proteomics‐integrated lipidomics	[237]
9	PknG kinase	Regulates host–pathogen interactions and inhibits phagosome‐lysosome fusion.	Phosphoproteomics	[238]
10	DosR regulon proteins	Helps MTB adapt to hypoxic conditions during latent infection, making them potential targets for latency control.	Differential proteomics	[239]
11	Oxidative stress response proteins	Include peroxiredoxins and catalases, which neutralize host‐induced oxidative damage to MTB.	Comparative proteomics	[240]
12	PE/PPE family proteins	Modulate host immune responses and contribute to antigenic variation and immune evasion.	Structural proteomics	[241]
13	RpoB	Mutation in RpoB leads to RIF resistance, making it a critical target for new drug development.	Proteomic profiling of resistant strains	[242]
14	Trehalose dimycolate synthesis enzymes	Contribute to MTB's cell wall formation and immune evasion.	Functional proteomics	[243]
15	KasA/KasB enzymes	It is key in mycolic acid synthesis and is essential for MTB cell wall structure.	Label‐free quantitation proteomics	[244]
16	EmbCAB complex	Involved in arabinogalactan biosynthesis, a crucial component of the mycobacterial cell wall.	Isobaric tagging proteomics	[245]
17	MmpL transport proteins	Participate in the transport of lipids necessary for MTB virulence and survival.	Shotgun proteomics	[246]
18	SigH sigma factor	Regulates the expression of stress response genes during host immune attack.	Differential proteomics	[247, 248]
19	EspE proteins	Facilitate host immune modulation, helping MTB evade detection.	Label‐free proteomics	[249]

Abbreviations: ESAT‐6, early secreted antigenic target‐6; PE/PPE, pro‐glu/pro‐pro‐glu family; RIF, Rifampicin; TB, tuberculosis.

**TABLE 7:** Development of microbiome‐based therapeutic proteins in TB treatment.

Sr. no	Therapeutic protein	Source (microbiome or engineered microbe)	Mechanism of action	Current status	Refs
1	Recombinant Mycobacterium proteins (e.g., Ag85 complex)	Engineered *Mycobacterium smegmatis*	Boosts host immunity by acting as a vaccine adjuvant and enhancing T‐cell responses.	Clinical trials as vaccine adjuvants in TB therapy	[251]
2	EsxA protein secretion inhibitors	Bacterial microbiome‐derived inhibitors	Blocks the EsxA protein secretion system to reduce virulence and immune evasion of MTB.	Early‐stage drug discovery pipeline	[252]
3	Resuscitation‐promoting factors	Dormant MTB and environmental microbiota	Revives dormant MTB to render it susceptible to conventional TB drugs.	Explored in conjunction with standard TB therapy to target latent infections	[253]
4	Human microbiota‐engineered peptides (e.g., LL‐37 mimetics)	Engineered gut commensals	Mimics natural antimicrobial peptides like LL‐37 to enhance bacterial clearance.	Preclinical studies in mouse TB models	[254]
5	Mycobacteriophage‐derived lysins	Engineered bacteriophages targeting MTB	Degrades MTB cell walls specifically, reducing bacterial load while sparing host microbiota.	Phase I clinical trials for safety and efficacy	[255]
6	Butyrate‐producing microbiota	GM (*Clostridium species*)	Enhances host immunity and regulates inflammation by promoting butyrate synthesis.	Potential adjunct in TB immunotherapy	[28]
7	Probiotic‐derived enzymes (e.g., superoxide dismutase)	Probiotic strains (e.g., *Lactobacillus reuteri*)	Protects host tissues from oxidative stress induced by MTB infection.	Experimental models showing reduced TB‐induced inflammation	[256]
8	Heat Shock Protein 65	Recombinant *Lactococcus lactis*	Boosts host T‐cell‐mediated immunity against MTB.	Preclinical vaccine candidate showing promise	[257]
9	Microbiota‐SCFAs (e.g., acetate, butyrate)	GM (e.g., *Bacteroides*)	Modulate immune responses by enhancing macrophage antimicrobial activity.	Explored to enhance the efficacy of TB vaccines	[258]
10	Siderophores (iron‐binding proteins)	Probiotic microbiota	Compete with MTB for iron, depriving the bacteria of a vital nutrient.	Investigated as part of combination therapies	[259]

Abbreviations: LL‐37, cathelicidin‐related antimicrobial peptide; TB, tuberculosis.

### Metabolomics approaches

Metabolomics, defined as the comprehensive analysis of metabolites (small molecules <50 Da) within a biological system, is a critical component of systems biology. It can show strong correlations with phenotypic changes, helping to understand physiological balance and how internal or external factors affect metabolic networks [261]. In GM research, it's useful for studying microbial activity and composition by quantifying GM‐related metabolites [262]. For instance, Folz et al. found that dietary and microbially derived metabolites contribute to metabolic differences among 15 healthy people [263]. It can also identify metabolic pathway alterations and track adaptive responses of the microbiota to environmental factors, including disease states [261, 264].

#### Techniques used in metabolomics

Metabolomic studies predominantly employ high‐throughput analytical platforms such as MS and nuclear magnetic resonance (NMR). MS‐based methods, including quadrupole MS and time‐of‐flight MS (TOFMS), are widely adopted due to their superior sensitivity, broad dynamic range, and capacity for multiplex metabolite detection [261, 262, 264]. Gas chromatography (GC)‐MS is good for analyzing primary metabolites, while LC‐MS is better for nonvolatile polar metabolites [261, 262, 264]. Capillary electrophoresis (CE) can separate charged small molecules efficiently [265]. Fernández‐García et al. [266] integrated GC‐quadrupole time‐of‐flight (QTOF)‐MS (volatile/acidic metabolites), LC‐QTOF‐MS (lipids), and CE‐TOFMS (positively charged metabolites) to characterize MTB‐induced metabolic reprogramming in murine lung tissue, uncovering novel host–pathogen interaction pathways.

Emerging MS imaging (MSI) techniques, such as matrix‐assisted laser desorption/ionization (MALDI) and desorption electrospray ionization, can directly show metabolite distribution in tissue sections [267, 268]. Prideaux et al. [269] applied MALDI‐MSI to study distributions of antitubercular drugs in infected lungs, and others correlated mycobacterial lipid marker localization with therapeutic efficacy [270].

NMR spectroscopy can identify metabolites with less sample preparation, although its sensitivity is lower. It is effective at detecting polar metabolites and can complement LC‐MS analyses. Andreas et al. [271] synergized NMR and LC‐MS to identify pediatric TB serum biomarkers, achieving high‐sensitivity detection of both lipophilic and hydrophilic metabolites.

The choice between untargeted and targeted metabolomics depends on experimental objectives. Untargeted approaches utilize high‐sensitivity instruments (e.g., LC‐TOFMS, GC × GC‐TOFMS) for global metabolite profiling [261]. For example, a GC × GC‐TOFMS study identified HIV/TB coinfection‐specific urinary metabolic signatures [272]. Targeted metabolomics, often employing LC‐MS or NMR, focuses on predefined pathways or metabolites. Building on prior untargeted findings [273], Isaiah et al. [274] used LC‐MS/MS to quantify GM‐associated organic acids in pediatric tuberculous meningitis (TBM) cases, establishing TBM‐specific metabolic dysregulation profiles.

**FIGURE 9:**
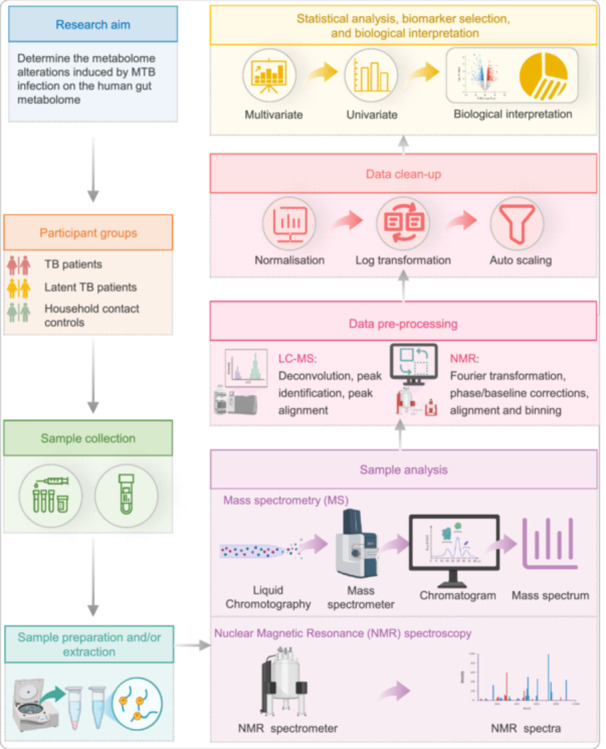
Overview of the general metabolomics workflow. The metabolomics pipeline comprises several key steps, beginning with sample collection and preparation, followed by analytical profiling using high‐resolution platforms such as chromatography, mass spectrometry (MS), nuclear magnetic resonance (NMR) spectroscopy. Data acquisition includes pre‐processing, clean‐up, and statistical analysis. The final step involves the identification and interpretation of metabolic signatures to reveal underlying biological processes and disease mechanisms. Some of the illustrative elements were created using BioRender (https://BioRender.com).

Data preprocessing varies for MS and NMR. MS data require peak deconvolution, alignment, and noise reduction [275, 276], while NMR data undergo Fourier transformation, spectral correction, and dimension reduction via binning [275]. Normalization and statistical analyses help find differential metabolites [275, 276]. Figure 9 summarizes a standardized metabolomics workflow. Building on these advanced analytical techniques, recent studies have applied metabolomics to unravel the complex interactions between the GM and TB.

#### Metabolome‐profiling in GM–TB interactions

MTB establishes its infectious niche by remodeling host metabolic networks, primarily disrupting lipid, carbohydrate, and amino acid homeostasis. Wang et al. [25] employed untargeted GC‐MS and 16S rRNA gene sequencing to demonstrate that active TB significantly depletes SCFAs and reshapes the fecal metabolome compared to HCs. SCFAs play dual roles in maintaining intestinal barrier integrity and modulating immune responses, and their reduction may exacerbate inflammatory cascades [25].

Beyond energy metabolism, amino acid dysregulation emerges as a critical feature of TB pathogenesis. TB patients show reduced serum levels of histidine and glutamine [277, 278], while perturbations in tryptophan, arginine, and gamma‐aminobutyrate (GABA) pathways directly influence immune signaling and host defense mechanisms [279]. Albors‐Vaquer et al. [280] further identified distinct serum metabolic signatures that differentiate active TB patients from asymptomatic household contacts and HCs, highlighting amino acid reprogramming as a biomarker of infection status.

Metabolomics provides unique insights into host‐microbiome crosstalk during TB progression. Mason et al. [273] utilized GC‐MS to identify cerebrospinal fluid biomarkers for pediatric TBM, while Yang et al. [281] integrated fecal amplicon sequencing with LC‐MS/MS metabolomics to distinguish TB, LTBI, and HCs. Notably, metabolomic approaches demonstrate exceptional diagnostic specificity: Chen et al. identified phenylalanylphenylalanine as a serum biomarker differentiating TB from lung cancer using ultra performance liquid chromatography (UPLC)‐QTOF/MS [282], and urinary metabolomic profiles effectively discriminate TB from non‐tuberculous mycobacterial infections [283].

Longitudinal metabolomic profiling can predict TB progression. Weiner et al. [284] identified trans‐African serum biosignatures predicting TB progression in household contacts. Luo et al. [121] developed fecal biomarker panels to stratify LTBI progression risk.

Metabolomics holds translational potential for treatment optimization. Sahu et al. [122] tracked partial GM restoration and metabolic normalization in TB patients during treatment, and Jeyanathan et al. [285] studied BCG vaccination effects. Lipidomic profiling by UPLC‐QTOF MS found potential treatment response biomarkers [277, 286]. Opperman et al. and Luies et al. identified GM‐derived metabolites related to treatment failure [123, 287].

Despite these advances, challenges persist in generalizing findings due to interindividual metabolic variability influenced by GM composition, diet, and environmental factors. Large‐scale validation across diverse cohorts remains essential before clinical translation [261]. Nevertheless, metabolomics—particularly when integrated with multi‐omics and ML—offers unprecedented insights into GM‐TB interplay, advancing diagnostic, prognostic, and therapeutic strategies.

### AI technologies

The 2024 Nobel Prize in Physics recognized Geoffrey Hinton and John Hopfield for pioneering neural network architectures that emulate neurobiological processes, while the Chemistry Prize honored David Baker, Demis Hassabis, and John Jumper for their AI‐driven breakthroughs in protein structure prediction. These accolades underscore AI's transformative role in decoding complex biological systems, including genomic and microbiome datasets, to advance personalized diagnostics and therapeutic strategies [288, 289].

#### AI in analyzing and interpreting complex GM data

GM research generates high‐dimensional, heterogeneous datasets that are hard to analyze with conventional analytical methods. Microbiome datasets from sequencing need preprocessing due to class imbalance and sparsity before applying AI for pattern recognition (Tables 8 and 9) [291, 292, 293, 294, 295, 296, 297, 298, 299, 300, 301, 302, 303].

**Table 8:** Open‐source resources for carrying out GM data analysis.

Category	Resource/database	Description	Link	Refs
Microbiome databases	EasyMetagenome	A user‐friendly and flexible pipeline for shotgun metagenomic analysis	https://github.com/YongxinLiu/EasyMetagenome	[290]
	HMP	Comprehensive datasets of microbial communities from various human body sites	https://hmpdacc.org/	[291]
	Gut feeling knowledge base	Database on GM–host interactions, including metabolites and disease associations	https://hivelab.biochemistry.gwu.edu/gfkbbaseline	[292]
	IMG	Comprehensive resources for microbial genome and metagenome analysis	https://img.jgi.doe.gov/	[293]
Metabolomics	HMDB	Database of metabolites, including those related to the GM	https://hmdb.ca/	[294]
	METLIN	Repository of metabolomic and lipidomic data, useful for gut microbial metabolite analysis	https://metlin.scripps.edu/landing_page.php?pgcontent=mainPage	[295]
Microbial interactions	KEGG	Database linking microbiome functional pathways to host health and disease	https://www.genome.jp/kegg/	[296]
	BioCyc	Collection of pathway/genome databases for microbial and host interaction	https://biocyc.org/	[297]
	STRING	Database for protein–protein interactions, including microbial proteins	https://string-db.org/	[298]
Clinical datasets	EBI metagenomics	A platform for analyzing and archiving metagenomic data, including GM	https://www.ebi.ac.uk/metagenomics	[299]
	GMrepo	Comprehensive database of human GM‐related studies and datasets	https://gmrepo.humangut.info/home	[300]
Taxonomic databases	Greengenes	Database for 16S rRNA gene sequences to study microbial taxonomy	https://rnacentral.org/expert-database/greengenes	[301]
	SILVA ribosomal RNA gene database project	High‐quality ribosomal RNA sequences database for microbial taxonomy analysis	https://www.arb-silva.de/	[302]
	RDP	Database for analyzing and comparing rRNA sequences	https://www.glbrc.org/data-and-tools/glbrc-data-sets/ribosomal-database-project	[303]
Disease‐specific resources	GutMDisorder	Database linking the GM to metabolic disorders	http://bio-computing.hrbmu.edu.cn/gutMDisorder/	[304]

Abbreviations: EBI, European Bioinformatics Institute; GM, gut microbiota; GMrepo, Human Gut Microbiota Research Database; GutMDisorder, Gut Microbiota‐Metabolic Disorder Database; HMP, human microbiome project; SILVA, Small subunit rRNA gene database; STRING, Search Tool for the Retrieval of Interacting Genes/Proteins.

**TABLE 9:** Summary of implementation for AI/ML with their motivations.

Motive	Use case	AI/ML model utilized	Refs
GM clustering	Clustering enterotype microbiome for pattern identification	K‐Means clustering, PCA	[305]
Predictive modeling	Colorectal cancer detection via identifying specific microbes involved in dysbiosis	RF, XGboost, SVM	[306]
Diagnostic application	Identifying the microbiome as a biomarker for anti‐TB drug‐induced inflammation	RF	[70]
	Identification of microbiome pattern linked to ATB‐DILI	RF, SVM	[307]
	Automated diagnosis of TB via analyzing chest X‐ray scans	RF	[308]
Treatment outcome	Predictive modeling to address loss to follow‐up in TB management	XGboost	[309]
	ML models to predict treatment unsuccess in PTB patient	Decision trees	[310]
Personalized treatment	Identifying new TB therapy combinations based on patients with MDR and nonresponsive patients.	Multi‐modal AI agent	[311]
	Early detection systems to detect adverse prognosis in patients receiving anti‐TB therapy	XGBoost, RF, MLP, light GBM, logistic regression, and SVM	[312]

Abbreviations: ATB‐DILI, antituberculosis drug‐induced liver injury; GBM, gradient boosting machine; GM, gut microbiota; ML, machine learning; MDR‐TB, multi‐drug‐resistant tuberculosis; MLP, multi‐layer perceptron; PCA, principal component analysis; RF, random forest; SVM, support vector machine.

#### Types of AI techniques used for GM data analysis

ML has two main types: supervised learning and unsupervised learning. Supervised learning employs labeled data to train models for classification or regression, like Support Vector Machines (SVM), random forest (RF), and Naïve Bayes, which help find microbial biomarkers and disease diagnostics. For instance, RF identified *Flavonifractor plautii* as a colorectal cancer (CRC)‐specific GM biomarker [313]. Unsupervised learning identifies intrinsic patterns in unlabeled data through clustering (K‐means, DBSCAN), association rule mining, or dimensionality reduction (principal component analysis [PCA], t‐SNE, UMAP). Knights et al. [305] applied K‐means clustering to define GM enterotypes, and Shi et al. [314] demonstrated that combining Bray–Curtis and UniFrac metrics optimizes clustering robustness across diverse datasets.

Deep learning (DL) can handle high‐dimensional data (e.g., genomics, metagenomics) through hierarchical feature extraction, although it suffers from interpretability issues, particularly in clinical applications. Novielli et al. [306] integrated SHapley Additive exPlanations (SHAP) values into CRC‐GM analyses to explain DL models [306, 315].

#### Resources for databases for GM analysis

Bioinformaticians investigating GM–host interactions can leverage a suite of specialized tools and databases tailored for multi‐omics integration (Table 8) [290, 291, 292, 293, 294, 295, 296, 297, 298, 299, 300, 301, 302, 303, 304]. Foundational frameworks such as the Human Microbiome Project (HMP) [291] provide standardized references for microbial community profiling. For streamlined data processing, platforms like EasyMetagenome offer user‐friendly pipelines for metagenomic analysis [290], while functional annotation is supported by curated databases including the Gut Feeling Knowledge Base and the Integrated Microbial Genomes (IMG) system [292, 293].

Metabolomic integration is facilitated by human metabolome database and METLIN, which host comprehensive metabolite annotations and spectral libraries [294, 295]. To decipher host‐microbe crosstalk, pathway‐centric platforms like KEGG [296], BioCyc [297], and STRING [298] enable systematic analysis of metabolic interactions and protein networks.

Clinical‐translational resources further bridge mechanistic insights to patient‐oriented applications. The European Bioinformatics Institute Metagenomics platform [299] and GMrepo [300] provide clinical metagenomic datasets, whereas taxonomic classification relies on reference databases such as Greengenes [301], SILVA [302], and the Ribosomal Database Project [303]. For disease‐specific investigations, GutMDisorder curates experimentally validated GM‐disease associations [304]. These resources, combined with advanced ML algorithms, enhance our ability to uncover microbiome signatures and translate them into actionable insights for TB management.

#### ML algorithms for pattern recognition in GM‐TB studies

**FIGURE 10:**
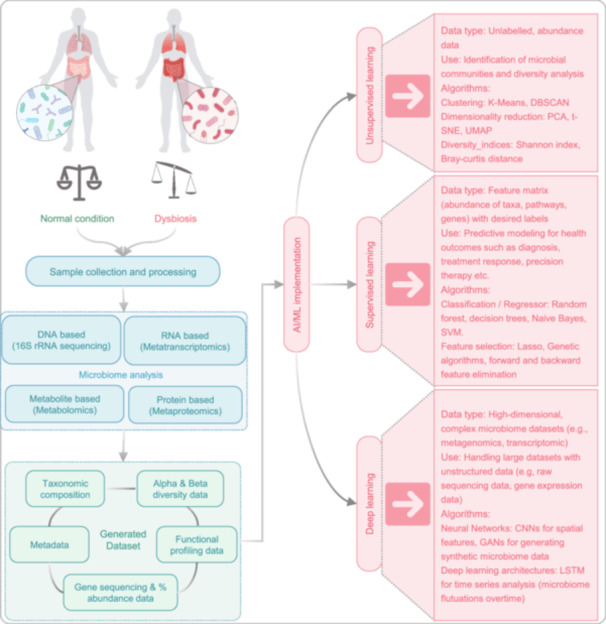
Applications of artificial intelligence (AI)/machine learning (ML) in microbiome research: from data collection to implementation. The diagram outlines the workflow for AI/ML integration in microbiome studies. The process begins with sample collection and processing, followed by multi‐omics profiling approaches (e.g., DNA, RNA, metabolite, and protein‐based analyses) to generate high‐dimensional datasets (e.g., taxonomic composition, alpha and beta diversity, functional profiling). Unsupervised learning methods, such as clustering and dimensionality reduction, are used to explore microbial community structure and diversity. Supervised learning algorithms (e.g., classification and regression) enable prediction of clinically relevant outcomes, such as disease diagnosis and treatment response. Deep learning (DL) methods, using architectures such as neural networks and Long Short‐Term Memory (LSTM) networks, further enhance the ability to model complex, nonlinear relationships within microbiome data.

ML has emerged as a pivotal tool for uncovering microbiome signatures in TB pathogenesis and treatment outcomes (Figure 10). Wipperman et al. [70] used RF regression to analyze multi‐modal data and found biomarkers for inflammatory resolution during anti‐TB therapy. Their model revealed inverse correlations between Bacilli/Pseudomonadota abundance and pro‐inflammatory cytokines (TNF‐α, IL‐6), demonstrating ML's capacity to decode microbiome–immune interactions. Furthermore, Wang et al. [307] addressed anti‐TB DILI (ATB‐DILI) through SVM and RF analysis of urine metabolomics from 69 patients. They identified *Negativicoccus* and *Actinotignum* as predictive taxa, achieving high diagnostic accuracy for ATB‐DILI risk stratification. Extending these principles to veterinary medicine, Lee et al. [316] developed an ML model with 96% accuracy for detecting *Mycobacterium avium* infections in cattle, pinpointing *Clostridium* as a key diagnostic marker. These studies collectively highlight ML's versatility in translating GM patterns into clinically actionable insights across human and animal TB research.

#### Predictive modeling for TB treatment response

##### Development of predictive models for treatment outcomes

Predictive modeling has emerged as a cornerstone in precision TB management, integrating multi‐dimensional patient data—including microbiome profiles, genetic markers, and immunological parameters—to forecast therapeutic trajectories. For treatment failure prediction, Peetluk et al. [310] developed a multivariate model identifying clinical and demographic predictors of unsuccessful pulmonary TB (PTB) outcomes. Addressing patient adherence, Chen et al. [309] employed XGBoost algorithms to predict loss to follow‐up risks in TB cohorts, enhancing intervention strategies.

Imaging‐driven prognostication further extends predictive capabilities. Lv et al. [308] demonstrated the utility of computed tomography (CT)‐derived radiomic features in forecasting sputum culture conversion timelines for MDR‐TB patients. Supervised learning applications have also advanced outcome stratification: Host et al. [317] applied supervised ML (RF, SVM, gradient‐boosted trees) to predict treatment success in DR‐TB, while Fayaz et al. [318] optimized decision tree algorithms for outcome prediction in PTB cohorts (AUC: 0.90, accuracy: 92.7%).

##### AI‐driven personalization of TB treatment strategies

Advances in microbiome analysis offer novel opportunities for precision medicine via host‐directed microbiota modulation. Probiotics, prebiotics, and microbiota transplantation could restore GM balance, serving as adjunct therapies tailored to individual GM profiles [127].

Multimodal AI systems that integrate clinical, imaging, and genetic data are enhancing predictive accuracy. Sambarey et al. [311] developed a model using multi‐domain data (radiological, microbiological, demographic) to predict MDR‐TB outcomes and find optimized drug combinations. Ford et al. [319] simplified complex antibiotic regimen design by training ML models on in vitro drug–pair interactions, enabling prediction of >500 higher‐order combinations to surpass current regimen efficacy. Liao et al. [312] created an AI tool for early detection of adverse prognoses (e.g., acute hepatitis, respiratory failure) in 2248 TB patients, demonstrating high AUCs for critical outcomes. Such models enable timely regimen adjustments, improving survival in high‐risk populations.

##### Predictive modeling for enhanced TB diagnostics

Traditional TB diagnostics (sputum smear microscopy, culture, and polymerase chain reaction) face limitations in sensitivity, speed, and resource demands. AI‐driven solutions are emerging to address these gaps (Table 9). Xiong et al. [320] developed TB‐AI, a CNN model detecting acid‐fast bacilli in microscopy images with 97.94% sensitivity and 83.65% specificity, reducing diagnostic delays and human error. Sharma et al. [321] applied AI to preprocessed chest X‐rays (histogram filtering, CLAHE, median filtering), achieving 98% accuracy with decision trees for pulmonary/extrapulmonary TB detection.

Cough audio analysis represents a promising low‐resource diagnostic frontier. Models like HeAR [322], Swaasa AI [323], and TBscreen [324] leverage self‐supervised learning on datasets from high‐burden regions. However, challenges remain in deploying complex models (e.g., Google's HeAR, trained on 313 million audio clips) on portable devices, which necessitates optimization via distillation or quantization [325].

#### Challenges and ethical considerations in AI‐driven TB research

The integration of AI into TB research and clinical practice offers transformative potential for improving diagnostics and treatment, yet it introduces critical technical and ethical challenges that demand urgent resolution [326]. A primary concern lies in data limitations and algorithmic bias, as current datasets often inadequately represent diverse global populations, compromising the reliability and generalizability of AI models—a challenge compounded by the “black box” nature of many systems, which undermines clinical trust and underscores the need for explainable AI (XAI) frameworks [327, 328, 329]. Equally pressing are ethical dilemmas surrounding data privacy, equity, and accountability, particularly given the risk of exacerbating global health disparities if AI tools are disproportionately trained on data from high‐income populations, leaving low‐resource, high‐TB‐burden regions underserved [330]. To address these interconnected issues, multidisciplinary collaboration is essential: policymakers must establish robust standards for data privacy and algorithmic auditing, researchers should prioritize creating open‐access, demographically diverse datasets while validating models across heterogeneous cohorts [331], and healthcare systems need to invest in clinician education to align AI capabilities with practical workflows. Only through such coordinated efforts—balancing technological innovation with ethical governance and infrastructural adaptation—can stakeholders overcome these barriers to fully harness AI's potential in reducing the global TB burden.

### Microbiome‐TB bioinformatics

Recent advances in sequencing technologies and bioinformatics methodologies [332, 333] have revolutionized microbiome research. However, they bring challenges in managing the scale and complexity of generated data. Microbiome bioinformatics is key to processing and analyzing these datasets in the context of GM‐TB dynamics [22, 334, 335]. This section explores key aspects of microbiome bioinformatics, including data management pipelines, multi‐omics integration, and specialized tools for GM‐TB research.

#### Data management and analysis pipelines

Effective microbiome‐TB research hinges on standardized workflows for managing heterogeneous datasets. Bioinformatics pipelines address challenges in data storage, computational processing, and analytical reproducibility, enabling researchers to gain insights from complex microbial communities.

##### Challenges in managing large‐scale microbiome data

High‐throughput sequencing generates voluminous and heterogeneous datasets, posing significant logistical and analytical hurdles. Storing and retrieving this data is difficult, and the variety of data types (like 16S rRNA profiling, shotgun metagenomics, metabolomics, and clinical metadata) makes integration complex due to format and analytical differences [290, 336, 337, 338, 339, 340].

Standardization remains a persistent challenge. Variability in experimental protocols, sequencing platforms, and preprocessing methods introduces batch effects that obscure biological signals [340]. Conventional normalization tools like Combat [341] and Limma [342] often fail to address the sparsity and high dimensionality of microbiome datasets. The absence of universal data standards and metadata reporting guidelines exacerbates these issues, hindering cross‐study comparisons and reproducibility [293].

Computational demands are high for microbiome data analysis, requiring high‐performance computing resources and specialized pipelines for quality control, taxonomic classification, functional annotation, and statistical modeling [343, 344]. Ethical considerations in data sharing, involving sensitive clinical information add to the complexity.

##### Development of bioinformatics tools for data analysis

Specialized tools address distinct stages of microbiome analysis (Table 10) [300, 323, 345, 346, 347, 348, 349, 350, 351, 352, 353, 354, 355, 356, 357, 358, 359, 360, 361, 362]. Quality control begins with FastQC [363], which evaluates raw sequencing quality, followed by Trimmomatic [364] for adapter removal and read trimming. DADA2 [323] and EasyAmplicon [347] enhance accuracy by resolving amplicon sequence variants, and distinguishing biological sequences from technical artifacts.

**TABLE 10:** Bioinformatics tools for data analysis.

Tool name	Function/application area	Key features	Refs
FastQC	Quality control and preprocessing	Provides comprehensive quality assessments of raw sequencing data, identifying issues like low base quality and adapter contamination.	[345]
Trimmomatic	Quality control and preprocessing	To enhance data reliability, perform trimming of low‐quality bases and remove adapter sequences.	[346]
DADA2	Quality control and preprocessing	Denoises sequencing data by distinguishing true biological sequences from sequencing artifacts.	[323]
EasyAmplicon	Amplicon data analysis	An easy‐to‐use, open‐source, reproducible, and community ‐based pipeline.	[347]
QIIME2	Taxonomic classification	Versatile platform for end‐to‐end microbiome analysis, integrating plugins for sequence processing, taxonomic assignment, and diversity analysis.	[348]
MetaPhlAn 4	Taxonomic classification	Profiles microbial communities using clade‐specific marker genes for rapid and precise taxonomic assignment.	[349]
Kraken2	Taxonomic classification	Employs ultra‐fast k‐mer‐based sequence classification for high‐resolution microbial profiling.	[350]
HUMAnN	Functional profiling	Reconstructs metabolic pathways and quantifies their abundance within microbial communities.	[351]
PICRUSt	Functional profiling	Predicts functional gene content based on phylogenetic placement of taxa, inferring metabolic potential from taxonomic profiles.	[352]
phyloseq(R package)	Statistical analysis and visualization	Comprehensive framework for importing, analyzing, and visualizing microbiome census data.	[353]
vegan (R package)	Statistical analysis and visualization	Advanced ecological analysis tools for examining diversity and community structure.	[354]
MicrobiomeAnalyst	Statistical analysis and visualization	Web‐based platform offers a user‐friendly interface for statistical testing, diversity analysis, and data visualization.	[355]
MetaWARP	High‐throughput data processing	Accelerates workflows for large‐scale metagenomic data analysis.	[356]
UPARSE	OTU clustering	Improves taxonomic assignment and reduces error rates in microbiome data analysis.	[357]
Mothur	16S rRNA sequence processing	Provides sequence processing, alignment, and analysis tools in microbial ecology studies.	[358]
Parallel‐META3	Functional annotation	High‐throughput tool for large‐scale metagenomic data set analysis	[359]
MTBseq	WGS analysis	Integrates various analytical steps into a user‐friendly framework for TB research	[360]
VSEARCH	OTU clustering	Improves taxonomic assignment and reduces error rates in microbiome data analysis.	[361]
GMrepo v2	Microbiome database	Facilitates storage and querying of microbiome datasets for comparative analysis.	[300]
USEARCH12	OTU picking and clustering	Speeds up OTU assignment and enhances data pre‐processing.	[362]

Abbreviation: WGS, whole‐genome sequencing.

Taxonomic profiling leverages platforms like QIIME2 [365], which integrates plugins for end‐to‐end analysis, and MetaPhlAn4 [349], which uses clade‐specific marker genes for rapid classification. Kraken2 [366] employs k‐mer matching for high‐resolution taxonomic assignments. TB‐specific tools such as MTBseq [360] enable WGS of MTB, while MUBII‐TB‐DB [367] tracks drug resistance mutations critical for TB control.

Functional insights are derived via HUMAnN [368], which reconstructs metabolic pathways from metagenomic data, and PICRUSt [369], which infers gene content from taxonomic profiles. Downstream analysis employs R packages (phyloseq, vegan) and MicrobiomeAnalyst [370], a web platform for statistical testing, differential abundance analysis, and visualization. These tools collectively enable robust characterization of microbial communities and their functional roles in TB.

#### Integration of multi‐omics data

TB pathogenesis involves intricate interactions between host genetics, immune responses, and GM dynamics. Multi‐omics integration—combining genomics, transcriptomics, proteomics, and metabolomics—provides a systems‐level understanding of these interactions, identifying biomarkers and mechanistic pathways (Table 11) [371, 372, 373, 374, 375, 376, 377, 378, 379, 380, 381, 382, 383, 384, 385, 386].

**TABLE 11:** Overview of multi‐omic data preprocessing and integration.

Name	Function/application area	Key features	Refs
MultiQC	Data aggregation and reporting	Aggregates results from various bioinformatics analyses into a single report, facilitating comprehensive multi‐omics data visualization.	[371]
MetaboAnalyst	Multi‐omics data integration and analysis	It provides tools for metabolomic data analysis, including normalization, statistical testing, and pathway analysis, and it is integrated with other omics data.	[372]
iCluster	Integrated clustering of multi‐omics data	Enables simultaneous clustering of multiple omics datasets, identifying shared patterns and associations across different molecular layers.	[373]
MixOmics	Multi‐omics data integration and visualization	Offers a suite of statistical methods for integrating and visualizing relationships between different omics datasets, including PLS‐DA and correlation analysis.	[374]
MOFA	Dimensionality reduction for multi‐omics data	Implements factor analysis to identify latent factors that capture shared and unique variations across multiple omics layers, enhancing data interpretation.	[375]
OmicsNet	Network‐based integration of multi‐omics data	Constructs interaction networks that integrate genomic, transcriptomic, and proteomic data, highlighting key nodes and pathways involved in biological processes.	[376]
Cytoscape	Network visualization and analysis	Facilitates the visualization and analysis of complex interaction networks, integrating multi‐omics data to identify functional relationships and network hubs.	[377]
Galaxy	Integrative omics pipelines	An open‐source platform that supports the construction of custom workflows for integrating and analyzing multi‐omics data using a wide range of bioinformatics tools.	[378]
Qiita	Microbiome data management	Facilitates organization, sharing, and analysis of large‐scale microbiome datasets.	[379]
MG‐RAST	Metagenomic data integration	Provides a comprehensive pipeline for analyzing and integrating metagenomic data, including functional annotation and taxonomic classification across multiple datasets.	[380]
MetaPhlAn	Microbial community profiling	Provides taxonomic profiles, aiding in the characterization of microbial communities.	[381]
NetMoss	Network module structure shift analysis	Using integrated multi‐omics data, evaluating shifts in network modules across different states, and identifying key bacteria and interactions associated with disease transitions.	[382]
WGCNA	Network construction and module detection	Constructs weighted gene co‐expression networks, facilitating the identification of modules and hub genes across multi‐omics datasets.	[383]
IMP	Multi‐omics integration	Facilitates comprehensive analysis of microbiome‐host interactions.	[384]
EBI‐metagenomics	Metagenomics analysis	Provides pipelines for functional and taxonomic annotation of metagenomics data	[299]
Enrichr	Gene set enrichment analysis	Provides easy‐to‐use pathways and gene‐set enrichment analysis, enabling functional annotation and biological interpretation.	[385]
OmicsIntegrator	Network‐based multi‐omics integration	Maps protein data onto interactome networks and identifies high‐confidence subnetworks relevant to the data. Uses the Prize‐Collecting Steiner Forest algorithm to discover cellular pathways and protein interactions	[386]

Abbreviation: PLS‐DA, partial least squares‐discriminant analysis.

##### Combining genomics, transcriptomics, proteomics, and metabolomics data

**FIGURE 11:**
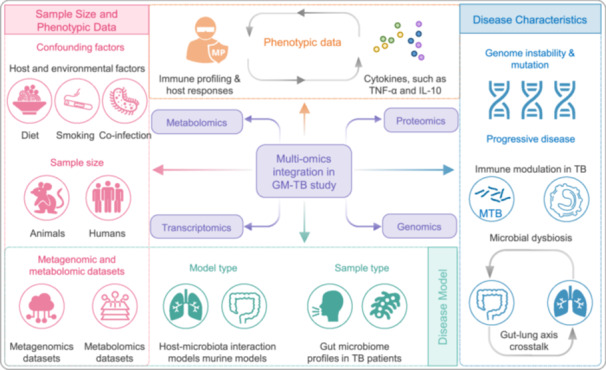
Integrative multi‐omics framework for investigating gut microbiota‐tuberculosis (GM–TB) interactions. This schematic illustrates a comprehensive strategy for multi‐omics integration in GM‐TB research, combining metabolomics, proteomics, transcriptomics, and genomics approaches. The framework incorporates key study parameters, including sample size, phenotypic data, disease models, and clinical or experimental disease characteristics, to facilitate a systems‐level understanding of TB pathogenesis. Central themes include genomic instability and mutations, immune modulation, microbial dysbiosis, and the mechanistic interplay between the lung and gut microbiota.

The systematic integration of multi‐omics data (spanning genomics, transcriptomics, proteomics, and metabolomics) is critical for elucidating the complex biological networks governing host–microbe interactions in TB and GM dynamics [387, 388, 389]. This holistic approach begins with rigorous data alignment across omics layers to ensure biological relevance and analytical coherence, followed by normalization techniques (e.g., z‐score or quantile normalization) to mitigate technical variability and preserve biologically meaningful signals. To further streamline complexity, dimensionality reduction methods such as PCA [390], t‐SNE [391], and gene panel selection algorithms [392, 393] enhance the identification of cross‐omics correlations critical to GM‐TB interplay, ultimately enabling the construction of comprehensive molecular maps that reveal metabolic pathways and functional relationships between GM and MTB (Figure 8). Genomic studies, leveraging high‐throughput sequencing, uncover the genetic basis of host–pathogen interactions, including reduced microbial diversity and antibiotic resistance determinants in TB patients [394, 395], while transcriptomics captures dynamic gene expression patterns such as upregulated pro‐inflammatory cytokines (e.g., IL‐6, TNF‐α) and microbiota‐derived genes in TB pathogenesis [396, 397]. Complementing these layers, proteomics characterizes protein networks and posttranslational modifications linked to immune signaling and MTB survival [189]. Metabolomics further enhances this systems‐level understanding by profiling host and pathogen metabolic changes, revealing altered metabolism and immune‐related metabolite signatures that could reflect both TB severity and treatment response [122, 261, 262, 263, 264]. Finally, computational integration tools like MiBiOmics, DIABLO, and IMP correlate microbial metabolites with host gene expression and protein activity, thereby unraveling pathogenic pathways and identifying potential intervention strategies (Figure 11) [398].

##### Multi‐omics approaches to deciphering GM–TB interactions

Multi‐omics methodologies are indispensable for unraveling the complex interplay between GM and MTB pathogenesis [23], enabling systematic dissection of host–microbe interactions that orchestrate immune responses to TB. For instance, correlating host transcriptomic profiles with microbial genomic data identifies specific taxa that modulate immune‐related gene expression, with SCFAs emerging as pivotal mediators in this crosstalk [26, 399, 400]. Notably, GM dysbiosis in TB patients disrupts SCFA synthesis, impairing cytokine pathways critical for MTB containment [401, 402, 403], while metagenomic–metabolomic integration uncovers microbial metabolic pathways driving host–microbe biochemical exchanges, thereby highlighting therapeutic targets [404, 405, 406].

Beyond mechanistic insights, multi‐omics integration enables biomarker discovery: proteomic studies detect microbial enzymes (e.g., SCFA‐producing butyrate kinase) and host proteins linked to granuloma formation, whereas transcriptomic analyses reveal immune‐regulatory genes (e.g., IL‐10, TGF‐β) associated with inflammation resolution [405, 406, 407], collectively informing diagnostic tools and personalized therapies. To synthesize these multidimensional data, computational frameworks like network analysis map interactions between microbial taxa and host immune pathways, identifying hub nodes (e.g., SCFA‐regulated cytokine networks) governing MTB containment [408], while predictive models simulate how perturbations (e.g., antibiotics, diet) alter GM‐TB dynamics, guiding hypothesis‐driven interventions. Crucially, the gut–lung axis serves as a pivotal interface in TB pathogenesis, where multi‐omics elucidates how GM dysbiosis impairs lung immunity via SCFAs and gut‐derived metabolites [32, 40, 409, 410, 411]. Supporting this, metabolomic studies demonstrate that gut‐derived metabolites reshape the lung microenvironment by modulating immune cell recruitment and cytokine balance, positioning GM‐targeted restoration of SCFA production as a promising strategy to enhance anti‐TB immunity.

#### Bioinformatics tools for GM‐TB research

Bioinformatics plays a pivotal role in addressing the analytical challenges posed by complex datasets in GM‐TB research. Table 12 summarizes essential bioinformatics resources critical to GM‐TB studies, emphasizing their functionalities in managing, analyzing, and interpreting microbiome data [296, 367, 412, 413, 414, 415, 416, 417, 418, 419, 420, 421].

**TABLE 12:** Resources, databases, and platforms for GM‐TB research.

Name	Function/application area	Key features	Refs
HMP	Microbiome database	Comprehensive data on human microbiota across various body sites serve as a comparative study reference.	[412]
IMG/M	Microbiome database	Repository for metagenomic datasets, enabling access and analysis of microbiome data relevant to TB studies.	[413]
TB Database	TB‐Specific database	Curated genomic, transcriptomic, and proteomic data specific to MTB and related strains.	[414]
Mycobrowser	TB‐Specific database	The platform for exploring Mycobacterium species' genomic and functional annotations facilitates host‐microbe studies.	[415]
OmicsDI	Multi‐omics integration platform	Integrates various omics datasets, allowing comprehensive analyses involving genomics, transcriptomics, proteomics, etc.	[416]
MetaboAnalyst	Multi‐omics integration platform	Supports metabolomics data analysis and integrates with other omics data types for holistic metabolic interaction studies.	[417]
KEGG	Functional annotation tool	Provides pathway maps and functional annotations to understand metabolic and signaling pathways in GM–TB interactions.	[296]
SEED SILAC or TMT	Functional annotation tool	Framework for annotating and analyzing genomic and metagenomic data, exploring functional capabilities of microbial communities.	[418]
MUBII‐TB‐DB	Antibiotic resistance	Documents mutations associated with resistance in MTB.	[367]
COMBAT‐TB Workbench	Sequence analysis	Provides tools and workflows for variant discovery and phylogenetic analysis for TB research	[419]
GTEx Project	Tissue‐specific expression database	Comprehensive resource for studying tissue‐specific gene expression and linking it to microbiota–TB interactions.	[420]
ConnectivityMap	Gene expression data repository and analysis platform	Houses a perturbation‐driven gene expression data set for discovering biological connections and analyzing gene expression signatures	[421]

Abbreviations: GM‐TB, gut microbiota‐tuberculosis; KEGG, kyoto encyclopedia of genes and genomes.

##### Specialized bioinformatics resources and databases

Specialized databases and analytical platforms are indispensable for advancing GM‐TB research. Microbiome repositories such as the HMP [291] and IMG (IMG/M) [293, 413] provide foundational datasets for comparative studies. HMP catalogs microbiota across human body niches, serving as a reference for GM‐TB investigations, while IMG/M offers metagenomic datasets with curated annotations for contextualizing microbial roles in TB pathogenesis. TB‐focused databases like the TB Database [414, 422] and Mycobrowser [415] centralize genomic, transcriptomic, and proteomic data on MTB strains, enabling comparative analyses of virulence and host adaptation.

Platforms such as Qiita [379] and EBI Metagenomics streamline meta‐analyses of multi‐omics datasets, supporting taxonomic and functional profiling of TB‐associated GM. Tools like PICRUSt predict microbial functional potential from metagenomic data, linking metabolic pathways to host immunity and TB progression. Proteomic interactome resources, including SInCRe [423], map host–pathogen protein interactions, elucidating molecular mechanisms of MTB persistence.

Multi‐omics integration platforms (e.g., OmicsDI [424], MetaboAnalyst [417, 425]) synthesize genomics, transcriptomics, and metabolomics data, revealing metabolic exchanges between GM and host systems. Emerging tools like MOSCA 2.0 [426] and gNOMO [427] enhance analytical precision through spectral clustering and biomarker selection algorithms. Functional annotation resources such as KEGG [296] and SEED [418] decode microbial pathways, while STRING [298] maps protein interaction networks critical to host‐MTB cross‐talk.

Databases like PATRICK [428] and GMTV [429] integrate multi‐omics data with clinical metadata, tracing genomic variations in MTB virulence and DR. Advanced tools such as MiBiOmics [430] and MultiDGD [431] employ DL to model transcriptome–metabolome interactions, identifying dysbiosis‐driven TB regulatory mechanisms. Platforms focused on vaccine development (e.g., MtbVeb [432], TBDReaM [433]) catalog immunogenic targets and DR mutations, bridging computational insights with therapeutic design. The addition of platforms such as MAGqual [434] and MetaWRAP [356] facilitates efficient data processing and analysis, increasing the accuracy and comprehensiveness of GM‐TB research.

##### Training and collaborative support for researchers

The effective application of bioinformatics tools in microbiome research hinges on comprehensive training programs and robust support systems tailored to researchers' diverse expertise levels. Proficiency in managing large‐scale omics data and addressing challenges associated with non‐standardized metadata requires specialized education for both bioinformaticians and experimental biologists [435, 436]. Institutions such as EBI [437, 438] and online learning platforms like Coursera and edX offer structured courses covering essential bioinformatics concepts, genomic analysis techniques, and multi‐omics integration strategies specifically adapted for microbiome studies. These programs are complemented by practical training through webinars and workshops that provide hands‐on experience with widely used analytical platforms including QIIME2 [365], PICRUSt [352], MiBiOmics [430], and MetaboAnalyst. Such interactive sessions enable researchers to master data processing workflows and optimize tool application for their specific datasets.

To facilitate independent analysis, major resources listed in Tables 10, 11, 12 incorporate detailed documentation, video tutorials, and active user communities that demystify complex analytical procedures. The open‐source paradigm further enhances accessibility through freely modifiable codebases and collaborative development frameworks [439]. Platforms like GitHub serve as critical repositories for sharing analytical workflows and experimental datasets, while international consortia such as the Global Microbiome Consortium and TBPortals Network foster interdisciplinary collaboration between microbiologists, computational biologists, and clinicians [440]. Specialized forums including BioStar, SEQanswers [441], and the Stack Overflow OMICS Forum create vital spaces for troubleshooting, knowledge exchange, and dissemination of best practices. Emerging platforms like KBase [442] extend these capabilities by enabling collaborative workflow development and transparent method sharing.

While these educational and collaborative resources form a critical foundation, equitable access to computational infrastructure remains paramount. Cloud‐based solutions such as Amazon Web Services and Google Cloud provide essential scalable environments for processing large microbiome datasets, ensuring researchers without institutional high‐performance computing resources can still conduct sophisticated analyses. This integrated ecosystem of training, open‐source tools, collaborative platforms, and cloud infrastructure collectively drives innovation in GM‐TB research by lowering technical barriers and accelerating methodological advancements.

## CURRENT CHALLENGES AND FUTURE DIRECTIONS IN GM‐TB RESEARCH

While substantial advancements have been made in elucidating the interplay between GM and TB, this field remains nascent, confronting multifaceted challenges that hinder translational progress. Addressing these limitations is critical for refining research methodologies, interpreting findings accurately, and guiding clinical applications. This section systematically examines key obstacles in current research and proposes actionable strategies to advance the field.

### Key challenges and limitations

**FIGURE 12:**
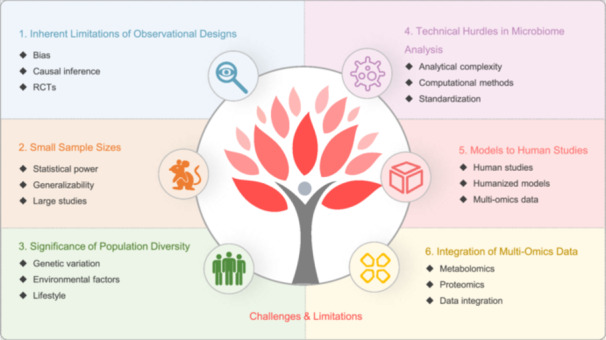
Challenges and Limitations in gut microbiota‐tuberculosis (GM‐TB) Studies. Current GM‐TB research faces several key challenges and limitations. Observational study designs limit the ability to infer causality, in contrast to the more rigorous evidence provided by randomized controlled trials (RCTs). Small sample sizes weaken statistical power and restrict the generalizability of findings. Population heterogeneity, including genetic background, environmental exposures, and lifestyle factors, is often underrepresented. Technical limitation in microbiome analysis, such as analytical complexity, inconsistence in computational methods, and the lack of standardization, further complicate data interpretation. Translational gaps between preclinical models and human studies remain a significant barrier. Lastly, the integration of multi‐omics data, including metabolomics and proteomics, poses analytical challenges due to differences in data structure, scale, and dimensionality.

Current GM‐TB research faces six major challenges (Figure 12). First, observational studies dominate the field, limiting causal inference due to potential confounding factors [66, 443]. While these studies identify associations between GM dysbiosis and TB outcomes, they cannot establish causality without controlled experimental designs. Second, small sample sizes in existing studies reduce statistical power and compromise the generalizability of findings, particularly when analyzing high‐dimensional omics data [444]. Third, population‐specific variations in GM composition—driven by genetic, geographic, dietary, and socioeconomic factors—restrict the external validity of regionally constrained studies [445, 446]. Fourth, technical limitations persist in microbiome characterization, including inconsistent sequencing protocols, bioinformatics pipelines, and data normalization methods, which hinder cross‐study comparisons [447]. Fifth, translational gaps exist between animal models and human pathophysiology [448], as murine models fail to fully replicate human TB progression or GM–host interactions. Finally, integrating multi‐omics datasets (metagenomics, metabolomics, transcriptomics) remains technically challenging, requiring advanced computational frameworks to disentangle complex GM–TB relationships.

### Strategic priorities for future research

**FIGURE 13:**
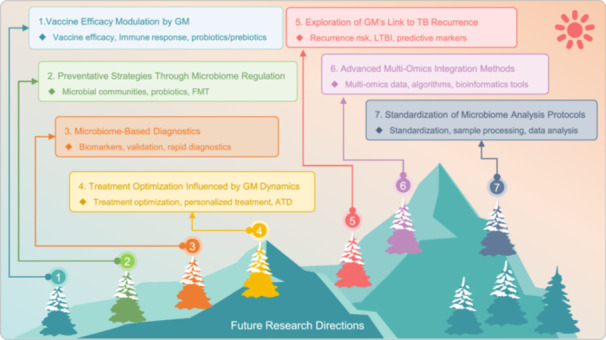
Future directions and research priorities in gut microbiota‐tuberculosis (GM‐TB) studies. Future research directions in GM‐TB studies encompass elucidating the role of GM in modulating vaccine efficacy and shaping host immune responses, along with exploring the therapeutic potential of probiotics, prebiotics, and FMT in TB prevention. Efforts are also directed towards developing microbiome‐based diagnostic tools by identifying and validating microbial biomarkers and creating rapid, noninvasive diagnostic platforms. Treatment optimization is a growing focus, emphasizing the need to consider GM dynamics to support personalized therapeutic approaches, including tailored antimicrobial regimens. Understanding the contribution of the GM to TB recurrence and latent tuberculosis infection (LTBI) is also critical, with research aimed to identifying predictive markers and recurrence risk factors. Advanced multi‐omics data integration is being facilitated through the development of sophisticated algorithms and bioinformatics tools. Lastly, standardization of microbiome analysis workflows, spanning sample processing and computational analysis, is crucial to ensure reproducibility, comparability, and translational relevance across studies.

To address these challenges, seven research priorities emerge (Figure 13). First, mechanistic studies should investigate GM‐mediated modulation of TB vaccine responses, including how specific microbial taxa or metabolites influence antigen presentation and adaptive immunity [449]. Second, longitudinal cohort studies are needed to evaluate GM's prophylactic potential, such as identifying protective microbial signatures or testing FMT in high‐risk populations [450]. Third, biomarker discovery efforts must prioritize validating GM‐derived diagnostic and prognostic signatures across diverse populations using standardized multi‐omics platforms [451]. Fourth, clinical trials should assess whether GM profiling can predict treatment outcomes, optimize anti‐TB drug regimens, or reduce adverse effects. Fifth, the underexplored link between GM dynamics and TB recurrence warrants investigation, particularly in posttreatment microbiological and immunological monitoring. Sixth, methodological advancements are required to harmonize multi‐omics data integration, including ML frameworks to resolve spatial–temporal interactions between GM, host immunity, and MTB. Lastly, international consortia should establish standardized protocols for GM analysis, encompassing sample collection, sequencing, and computational workflows to enhance reproducibility and cross‐study validation.

By systematically addressing these challenges and priorities, researchers can bridge critical knowledge gaps and accelerate the development of microbiome‐targeted interventions for TB prevention, diagnosis, and therapy.

## CONCLUSION

This review comprehensively explores the complex relationship between GM and TB, revealing significant findings and potential applications in this emerging field. Based on a systematic analysis of existing literature, we have drawn the following key conclusions:

Firstly, the GM composition in TB patients significantly differs from that in healthy individuals, characterized by a marked reduction in microbial diversity, changes in the abundance of specific genera, and imbalances in functional microbial communities. These changes reflect the impact of TB on the host's GM and may play a crucial role in the disease's onset, progression, and prognosis.

Secondly, the GM participates in TB's pathological processes through various mechanisms, including regulating host immune responses, influencing the growth and metabolism of MTB, and participating in drug metabolism. Notably, the regulatory role of the GM on the immune system may be a key link between GM and TB, providing new insights into the immune pathogenesis of TB.

Thirdly, the GM shows great potential in diagnosing, treating, and preventing TB. Microbiome‐based diagnostic methods may enhance the accuracy and timeliness of TB diagnosis. Strategies to improve TB treatment by modulating the GM and reducing adverse drug reactions are being explored. Additionally, probiotic supplementation or dietary interventions to prevent TB show promising potential.

Fourthly, applying new technologies in GM‐TB research opens new avenues for understanding and managing the disease. Advanced molecular techniques, such as NGS and metagenomics, enable more detailed and precise characterization of GM. ML and AI are also being applied to identify predictive biomarkers and develop more sophisticated diagnostic and prognostic models. These technological advances are crucial for translating basic research findings into clinical applications and improving patient outcomes.

However, current research faces several limitations, such as the constraints of observational studies, insufficient sample sizes, and population or regional differences. These limitations caution us to carefully interpret and apply research findings, and more high‐quality studies are needed to validate these results. Looking ahead, many areas in the GM‐TB research field warrant further exploration. These include studying the relationship between GM and TB vaccine efficacy, developing microbiome‐based diagnostic and prognostic tools, exploring the application of GM modulation in TB prevention and treatment, and leveraging new technologies to deepen our understanding of the GM–TB interaction. Particularly, large‐scale, long‐term prospective studies will help us better understand the role of the GM in TB's natural history.

In conclusion, research on GM and TB reveals profound connections Between the human microbiome and this ancient disease. As research deepens and technologies advance, we have reason to believe that new strategies based on GM will provide novel tools and insights for TB control, ultimately contributing to more effective management of this global public health issue.

## AUTHOR CONTRIBUTIONS

**Yanhua Liu**: Writing—original draft; visualization; methodology; investigation. **Ling Yang**: Investigation; writing—original draft; visualization; methodology. **Maryam Meskini**: Investigation; writing—original draft; methodology. **Anjana Goel**: Investigation; writing—original draft; visualization. **Monique Opperman**: Investigation; writing—original draft; methodology. **Sagar Singh Shyamal**: Investigation; writing—original draft; methodology. **Ajay Manaithiya**: Investigation; writing—original draft; methodology. **Meng Xiao**: Investigation; writing—original draft; methodology. **Ruizi Ni**: Investigation; methodology. **Yajing An**: Investigation; methodology. **Mingming Zhang**: Methodology; investigation. **Yuan Tian**: Investigation; methodology. **Shuang Zhou**: Investigation; methodology. **Zhaoyang Ye**: Investigation; methodology. **Li Zhuang**: Investigation; methodology. **Linsheng Li**: Investigation; methodology. **Istuti Saraswat**: Investigation; methodology. **Ankita Kar**: Investigation; methodology. **Syed Luqman Ali**: Investigation; methodology. **Shakir Ullah**: Investigation; methodology. **Syed Yasir Ali**: Investigation; methodology. **Shradha Kaushik**: Investigation; methodology. **Tianmu Tian**: Investigation; methodology. **Mingyang Jiao**: Investigation; methodology. **Shujun Wang**: Investigation; methodology. **Giulia Ghisleni**: Investigation; methodology. **Alice Armanni**: Investigation; methodology. **Sara Fumagalli**: Investigation; methodology. **WenYu Wang**: Investigation; methodology. **Chao Cao**: Investigation; methodology. **Maria Carpena**: Investigation; methodology; visualization. **Miguel A. Prieto**: Conceptualization; writing—original draft; writing—review and editing; resources; visualization. **Antonia Bruno**: Conceptualization; writing—review and editing; writing—original draft; resources. **Chanyuan Jin**: Conceptualization; writing—review and editing; writing—original draft; resources. **Hanqing Hu**: Conceptualization; writing—review and editing; writing—original draft; resources. **Yuhang Zhang**: Conceptualization; writing—review and editing; writing—original draft; resources. **Ilse du Preez**: Conceptualization; writing—review and editing; visualization; resources. **Ashok Aspatwar**: Conceptualization; writing—review and editing; resources; writing—original draft. **Lingxia Zhang**: Conceptualization; writing—review and editing; methodology; investigation; writing—original draft. **Wenping Gong**: Conceptualization; writing—review and editing; supervision; visualization; project administration; investigation; writing—original draft; resources.

## CONFLICT OF INTEREST STATEMENT

The authors have declared no competing interests.

## ETHICS STATEMENT

No animals or humans were involved in this study.

## Data Availability

No new data were generated or analyzed in this review. Supplementary materials (graphical abstract, slides, videos, Chinese translated version, and update materials) may be found in the online DOI or iMeta Science http://www.imeta.science/.
